# Deciphering Deleterious nsSNPs in MUC16's SEA Domain: Structural and Functional Implications in Cancer Metastasis via Computational Analysis

**DOI:** 10.1111/jcmm.70633

**Published:** 2025-06-06

**Authors:** Muaz Faruque, Maisha Maliha Medha, A. M. U. B. Mahfuz, Md. Monirul Islam, Md Afjalus Siraj

**Affiliations:** ^1^ Pharmacy Discipline, Life Science School Khulna University Khulna Bangladesh; ^2^ Department of Biotechnology and Genetic Engineering, Faculty of Life Science University of Development Alternative Dhaka Bangladesh; ^3^ Department of Pharmacy, Faculty of Health Sciences Gono Bishwabidyalay Dhaka Bangladesh

**Keywords:** glycosylation, metastasis, MUC16, non‐synonymous single nucleotide polymorphisms, PolyPhen‐2

## Abstract

MUC16 ranks among the top three genes exhibiting the highest mutation frequencies in various cancer types. It encodes transmembrane mucins present in the epithelial linings of the ocular, respiratory, gastric and female reproductive systems, serving to protect and maintain mucosal surfaces. Overexpression of MUC16 contributes to the differentiation, proliferation, invasion and metastasis of cancer cells in ovarian, endometrial, pancreatic, colon, breast and non‐small‐cell lung cancers. In this study, we analysed the structural and functional effects of pathogenic and potentially harmful non‐synonymous single nucleotide polymorphisms (nsSNPs) of MUC16, employing a blend of computational algorithms. Initially, SNPs data for MUC16 were gathered from the Ensembl database and refined using computational tools (PROVEAN, SIFT, PolyPhen‐2, SNAP‐2, MutPred, I‐Mutant3.0 and MUpro) to isolate four final pathogenic SNP variants (L151P, Y144N, C111Y and D108Y). Through evolutionary conservation analysis, we determined that these mutational variants originate from a highly conserved and stable domain. Our findings particularly emphasise the Y144N variant as a potentially highly deleterious mutation situated in the SEA5 domain. This variant could significantly impact stability, overall flexibility, compactness, expansion, glycosylation ability and metastatic potential when compared to both the wild‐type and other mutant variants. In summary, these findings shed light on missense mutational variants, providing insights into the vast array of disease susceptibilities associated with MUC16's glycosylation process. This understanding could aid in the development of effective drugs for diseases linked with these mutations.

## 1 Introduction

Mucins, also known as CA125, represent glycosylated proteins serving as biomarkers due to their elevation in the blood of certain cancer patients, particularly those with ovarian cancer (Bast et al. 1998). These glycoproteins are comprised of oligosaccharide chains covalently linked to high molecular weight amino acid chains. Primarily synthesised by epithelial cells, Mucins play a critical role in shielding the human body against external physical and biological threats [1]. They serve as protective agents for the luminal surfaces of epithelium‐lined passages and ducts in the respiratory, reproductive and gastrointestinal systems, defending against pathogens [2]. These versatile glycoproteins are categorised into two structural classes: transmembrane Mucins (such as MUC1, MUC3A, MUC3B, MUC4, MUC12, MUC13, MUC15, MUC16, MUC17 and MUC20) and gel‐forming or secreted Mucins (including MUC2, MUC5AC, MUC5B, MUC6, MUC7, MUC8 and MUC19) [3].

While all MUCs share certain structural traits, each possesses a distinctive domain organisation, sequence, length and quantity of tandem repeat (TR) sequences [4]. Transmembrane MUC structures typically encompass three primary domains: the N‐terminal, TR and C‐terminal. Both the N‐terminal and TR domains are predominantly extracellular and heavily glycosylated [5]. The TR region is characterised by sequences rich in serine, threonine and proline, pivotal for their structural and functional integrity [6]. This region, hosting over 60 repeats with 156 amino acids each, is acknowledged as the binding site for specific antibodies targeting MUC16.

The C‐terminal domain of MUC16 comprises multiple extracellular SEA (Sea urchin sperm protein, Enterokinase and Agrin) domains, a transmembrane segment and a cytoplasmic tail containing potential phosphorylation sites. The structural framework of MUCs plays a crucial role by fostering intricate interactions among various signalling molecules in distinct domains, including growth factor receptors and intercellular adhesion molecules [7]. The extensive glycosylation and the presence of growth factor‐like C‐terminal domains in MUC16's structure contribute to modulating the microenvironment around the cell surface. This structural configuration also induces modifications in glycosylation patterns during malignant transformations, facilitating interactions with other surface receptors. These interactions promote cancer cell differentiation, proliferation, invasion and metastasis [8, 9].

Within the category of membrane‐bound MUCs, MUC16 stands out as the second‐longest human protein following the muscle protein titin, boasting a substantial length of 22,152 amino acids [10]. In breast cancer cases, it has been identified as overexpressed, occurring in approximately 54% of incidences. Ordinarily, MUC16 finds expression in various mucosal surfaces, including the ocular surface, respiratory system, gastric tract and female reproductive organs [11, 12]. However, its overexpression extends beyond breast cancer and encompasses several tumour types, such as ovarian carcinoma, endometrial, pancreatic, colon, breast and non‐small‐cell lung cancers [13]. Additionally, it is associated with various conditions like dry eye disease, Sjogren's syndrome [14], psoriasis [15], pulmonary tuberculosis [16] and endometriosis [17]. MUC16's role extends to promoting breast cancer cell proliferation through its interaction with JAK2. This interaction triggers the phosphorylation of STAT3, culminating in the co‐transactivation of c‐Jun [18, 19].

In this investigation, an in‐depth analysis of the nsSNPs within the MUC16 protein was performed. Various computational tools employing diverse algorithms were utilised to assess the detrimental effects caused by these mutations. Additionally, molecular dynamics simulations were employed to gain deeper insights into how these substitutions might influence the overall structure of the protein.

## 2 Methods

### 2.1 Retrieval of the Information of SNPs

Amino acid sequence of the human MUC16 protein in FASTA format was downloaded from the UniProt database (UniProt ID—Q8WXI7) [20]. Experimental crystal structure of a human MUC16 SEA domain was collected from Protein Data Bank (PDB) [21]. Related SNP information of the human MUC16 gene was collected using NCBI Variation Viewer [22] (Sayers et al. 2022). Nonsense, stop lost, splice acceptor, splice donor and UTR SNPs of the MUC16 gene along with their rs (reference SNP) IDs were retrieved from Variation Viewer applying the following filters: dbSNP under ‘Source database' menu, single nucleotide variant under ‘Variant type' menu and nonsense (stop gained)/stop lost/splice acceptor variant/splice donor variant/5 prime UTR variant/3 prime UTR variant under ‘Molecular consequence' menu. In addition, information about the missense SNPs was downloaded directly from the NCBI dbSNP database [23].

### 2.2 Determination of Functional SNPs Using Different In Silico Algorithms

The chosen SNPs underwent a screening process to assess potential deleterious effects using a blend of diverse computational tools employing different algorithms. Among these tools, mutations identified as deleterious were selected for further analysis, while the remaining SNPs were excluded. All collected missense SNPs underwent scrutiny through PolyPhen‐2 (Polymorphism Phenotyping version 2), a tool employing sequence and structure homology‐based analysis. This tool utilises eight sequence‐based and three structure‐based parameters and employs two datasets –HumDiv and HumVar –to create and test two distinct PolyPhen‐2 models. Evaluating the Naive Bayes posterior probability, PolyPhen‐2 calculates false‐positive and true‐positive rates to determine the likelihood of a mutation being pathogenic [24]. Based on predefined false‐positive rate (FPR) thresholds, the SNPs are categorised as ‘Probably Damaging’, ‘Possibly Damaging’ or ‘Benign’.

SNPs identified as potentially pathogenic by Polyphen‐2 underwent further assessment using sequence homology‐based computational tools: sorting intolerant from tolerant (SIFT) and PROtein Variation Effect Analyser (PROVEAN). These tools aimed to determine whether the missense mutations carried damaging functional consequences. SIFT [25] operates by scanning the query protein against a protein database to find similar protein sequences. It selects sequences with appropriate diversity, aligns them with natural nsSNPs, and then analyzes the amino acid composition at a specific position to compute a score [26]. The resulting SIFT score, ranging from 0 to 1, represents the normalised probability of observing the new amino acid at that position. Scores between 0 and 0.05 are generally indicative of potential protein function disruption and are considered pathogenic [27]. PROVEAN is another predictive tool that assesses how variations in protein sequence impact protein function. This tool forms clusters of BLAST hits sharing over 75% global sequence identity, calculates the average of the top 30 clusters from a supporting sequence, and generates a final score [28]. Mutations with a final score below −2.5 are predicted as ‘deleterious’, while those above are classified as ‘neutral’. Mutations commonly identified as pathogenic by both SIFT and PROVEAN databases were selected for subsequent analysis.

The missense SNPs flagged as potentially harmful through homology‐based approaches underwent further scrutiny using two supervised learning methods: SNAP2 and MutPred. These servers employ distinct supervised learning algorithms to detect disease‐associated missense SNPs based on diverse statistical principles. Screening for non‐acceptable polymorphism v2 (SNAP2) operates as a neural network‐based machine learning classifier, predicting the effects of functional nsSNPs by considering parameters such as protein structure and residue conservation within sequence families [29]. Inputting the MUC16 protein sequence in FASTA format as the query, SNAP2 generates scores by analysing protein sequences and lists of mutants for each replacement. Predictions fall into two categories: mutations with scores ranging from −100 to 0 are anticipated to be neutral, while scores between 0 and 100 indicate potential effects. MutPred2, on the other hand, is a machine learning software that integrates genetic and molecular data to probabilistically predict the pathogenicity of amino acid substitutions [30]. Besides offering a general pathogenicity prediction, it provides a ranking of specific molecular alterations that could impact functionality. Mutations with a score above 0.5 are predicted to be pathogenic, with the probability of pathogenicity increasing as the score escalates.

### 2.3 Analysis of Protein Stability

Amino acid substitutions can lead to alterations in protein stability. So, the protein stability of the deleterious mutations was analysed by I‐Mutant3.0 [31]. This tool is a support vector machine‐based predictor which requires protein sequence information to be input and uses a database for protein mutation to assess the effect of missense SNPs on the stability of protein. The change in protein stability is evaluated by calculating the DDG value i.e., the change in free energy.

MUpro (https://mupro.proteomics.ics.uci.edu/). A prediction regarding the impact of the mutation on protein stability, along with a confidence level. Stability is steady at a score close to 0. A score close to −1 indicates a significant degree of unreliability. A score close to +1 indicates strong confidence in greater stability [32].

### 2.4 Selection of Most Deleterious nsSNPs

The deleterious nsSNPs identified through the combined use of homology‐based and supervised learning‐based computational tools within the SEA5 domain of MUC16 were chosen for further examination. In the sequential numbering of SEA domains in MUC16, ranging from SEA1 to SEA16, the initial 12 SEA domains, starting from the N‐terminus, exhibit the highest sequence homology, while the subsequent 4 SEA domains, closer to the membrane, present more distinct characteristics. The selection of the SEA5 domain stems from its representation of MUC16 SEA domains overall, boasting over 80% homology with other SEA domains [21].

### 2.5 Conservation Analysis of Amino Acid Residues

The most severe mutations chosen underwent further examination using the Consurf server to pinpoint mutations occurring within the conserved segments of the protein. Within a gene, highly conserved regions are prone to housing mutations that trigger diseases. Changes in these conserved areas tend to disrupt the protein's three‐dimensional structure and functionality, consequently leading to diseases. The Consurf server employs various algorithmic alignment methods to scrutinise the amino acids within a specific protein, predicting its evolutionary status by comparing the protein sequence with homologous sequences. This tool categorises predictions across nine grades of the colour spectrum, signifying whether the analysed amino acid residue is variable, neutral or conserved within the protein [33].

Phylogenetic tree was constructed for the MUC16 SEA5 domain by using the sequences from UniProtKB residues 12,695–12,821, structure residue numbering 35–160 (7SA9_A chain) (‘UniProt: the universal protein knowledgebase in 2023’, 2023). MEGA 11 package [34] was run for building the tree applying 7 closest identified matches to human MUC16 SEA5 domain from UniProt BLASTp [35, 36] search. The tree was constructed using the bootstrap value of 1000 [37] and the maximum likelihood technique. *Puma concolor* (A0A6P6HAA3_PUMCO), *Callorhinus ursinus* (A0A3Q7NAQ7_CALUR), *Lynx pardinus* (A0A485P732_LYNPA), *Equus caballus* (A0A5F5PRM6_HORSE), *Chlorocebus sabaeus* (A0A0D9R602_CHLSB), *Odobenus rosmarus divergens* (A0A2U3VKQ7_ODORO), *Bos indicus* (A0A6P5C241_BOSIN) protein FASTA file was download from UniProt [38] (‘UniProt: the universal protein knowledgebase in 2023’, 2023). Then these FASTA files were further used to build the phylogenetic tree.

### 2.6 Modelling and Structural Validation

To explore structural stability and deviations, we acquired the three‐dimensional X‐ray crystal structure of the wild‐type (WT) MUC16 from the PDB (ID: 7SA9). Cleaning of the protein involved the removal of water molecules and natural ligands, with the exclusion of chain B. However, the PDB files of 7SA9_A were lacking Lys118, Ser119, Pro120 and Gly121. To address this, the absent amino acid residues were rectified, and a modelled structure of the WT (7SA9_A) was constructed using Simlab [38], focusing solely on the core SEA domain [Uniprot residues 12,695–12,821, corresponding to structural residue numbers 35–160 in chain A of 7SA9 (White et al. 2022)]. Subsequently, mutant variant structures were modelled utilising YASARA software (Version 22.8.22.W.64) [39]. Energy minimisation of both the WT and mutant proteins was conducted through YASARA [39]. Moreover, validation of the structures of MUC16 SEA5 domains for both the WT and mutant variants was performed using the PROCHECK server [40]. Furthermore, the grade of the modelled structures was identified by the Ramachandran Plot [41]. Because the Ramachandran plot aids in determining the residues with a defect in the stereochemistry when the parameters are out of the acceptable region [42].

### 2.7 Ligand Preparation

The NLG and OLG ligand structures were sourced from the PDB under the IDs 1GYA and 6ELC, respectively. To ensure consistency in atom homology, only chain A of these ligands was taken into account, and chain B was removed. Subsequently, energy minimisation of both NLG and OLG was carried out using YASARA software (Version 22.8.22.W.64) [39, 43, 44].

### 2.8 Protein‐Ligand Molecular Docking

The YASARA software (Version 22.8.22.W.64) facilitated the molecular docking process involving both WT and mutant proteins with NLG and OLG [39]. For executing the docking, the default forcefield and AutoDock algorithm were employed [45, 46]. The YASARA program determined the Gibbs free energy (ΔG, kcal/mol), with more positive energy values indicating stronger binding. The docking results estimate the binding energy between the interaction of the protein and ligand as well as the ligand's orientation and positioning in a binding site [47]. After analysing the data, complexes with high binding energies (kcal/mol) were selected to test the stability of protein‐ligand complexes using molecular dynamics modelling.

### 2.9 Protein–Protein Docking

The 3D structure of mesothelin (PDB ID: 8CX3) was employed as the receptor molecule. In the process, all chains, atoms and water molecules, except for chain A, were removed from the mesothelin structure. Simultaneously, the structures of both the WT and mutant proteins, along with their complexes with NLG and OLG, were loaded as ligand molecules. For molecular docking studies, the ClusPro 2.0 server was utilised. The docking grid was specifically generated around the active site of mesothelin encompassing amino acid residues 299–359 [48]. Notably, PIPER utilises an expression in the form of ‘E' to denote the interaction energy between two proteins.

$$E=w_{1}E_{\mathrm{rep}}+w_{2}E_{\text{attr}}+w_{3}E_{\text{elec}}+w_{4}E_{\text{DARS}}$$

Where, the attractive and repulsive contributions to the van der Waals (VdW) interaction energy are denoted by *E*_rep_ and *E*_attr_, respectively. Electrostatic energy stands for *E*_elec_. The main contributions of *E*_DARS_, a paired structure‐based potential, are desolvation contributions or changes in free energy caused by the loss of water molecules from the interface. The weights of the respective residues are determined by the coefficients *w*_1_, *w*_2_, *w*_3_ and *w*_4_.

### 2.10 Superimposition and Visualisation

The alignment feature within the Yasara software was employed to superimpose all the structures [49] Visual representation of interactive residues was facilitated through the BIOVIA Discovery Studio 2021 Client [50].

### 2.11 Molecular Dynamics Simulation

The MD simulation aimed to gain comprehensive insights into the deleterious nature of the selected nsSNPs. Initially, the three‐dimensional X‐ray crystal structure of MUC16 (PDB ID: 7SA9) was obtained from the PDB, while the mutant structures were generated and refined using BIOVIA Discovery Studio Visualizer [50]. The simulations were carried out employing the GROMOS96 43a1 force field within a triclinic simulation box (36 × 36 × 44 Å). The systems were solvated using simple point‐charge (SPC) water models and neutralised with 0.9% sodium chloride to mimic physiological ion concentrations. Prior to commencing the actual dynamics, an energy minimisation step utilising the steepest descent method (5000 steps) was performed to relax the molecular geometry. Subsequently, equilibration was conducted to simulate human physiological conditions (300 K, 1 bar, pH 7.4), following which the simulations ran for 100 ns. Trajectory files were analysed to extract information regarding root mean square deviations (RMSD), root mean square fluctuations (RMSF), radius of gyration (Rg), solvent‐accessible surface area (SASA) and hydrogen bonds for each mutant and the WT protein [51].

The MD simulations targeted the selected nsSNPs by utilising their docking complexes—comprising mutant structures alongside the WT MUC16 structure—interacting with NLG and mesothelin binding sites. YASARA software's (Version 22.8.22.W.64) dynamic program [39] in conjunction with the amber 14 force field [52] facilitated these simulations. Short‐range VdW and Coulomb interactions were explored within simulation cells, employing an 8 Å cutoff radius. The PME (particle‐mesh Ewald) method was used to handle long‐range electrostatic interactions [53]. The simulation cells were cubic, sized 8 Å larger than the combined docked complexes, and filled with water, employing periodic boundary conditions. Before commencing the dynamics, energy minimisation was executed using the steepest descent method (5000 steps) to relax the system's molecular geometry. Subsequent equilibration aimed to mirror human physiological conditions, running for 100 ns at 310 K temperature, pH 7.4. A timestep of 2 × 1.25 fs was employed. The trajectory analyses focused on evaluating RMSD, RMSF, Rg, SASA, molecular surface area (MolSA) and H‐bonds to gain insight into the systems' behaviours and interactions.

### 2.12 Principal Component Analysis (PCA)

The PCA method reduces multivariate energy factors to a low‐dimensional space, highlighting the variabilities in the MD trajectory data without specifically identifying them [54, 55]. Bond lengths, bond angles, dihedral angles, planarity, VdW energies and electrostatic energies (Coulomb) are examples of factors that relate to the structural and energetic information of complexes and aid in the visualisation of any differences between various groups [56, 57]. 100 ns simulation data of WT and mutant variants complexes with NLG and mesothelin were utilised for PCA analysis. The following method was used to organise the multivariate components in the X matrix and reduce them into a product of two new matrices:

$$X={T_{k}P}_{k}^{t}+E$$

Here, *k* is the number of components in the model, *E* is the unmodeled variance, *T*_*k*_ is the matrix of scores that appear for the relationship between the sample and each other and *P* ^*k*^ is the matrix of loadings that convey information about the relationship between the variables [57]. *GraphPad Prism 9* was used to calculate the PCA calculation.

## 3 Results

The complete workflow, databases and tools used to identify the deleterious SNPs on the SEA5 domain of human *MUC16* and their structural and functional consequences due to mutations are summarised in Figure S1.

### 3.1 Retrieval Data of MUC16 SNPs

**FIGURE 1:**
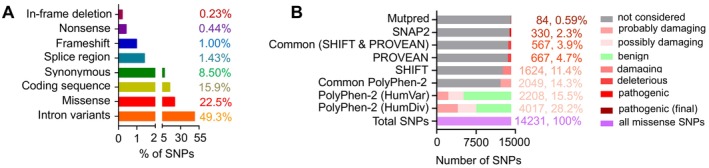
(A) Distribution of MUC16 intron variants, missense mutation, coding sequence, synonymous, splice region, frameshift, nonsense and inframe deletion. (B) Prediction results of the 84 pathogenic nsSNPs in the MUC16 analysed by the six computational tools.

The data regarding polymorphisms in the human MUC16 gene was obtained from the Ensembl GRCh37.p13 dbSNP database (Transcript ID: ENST00000397910.4), encompassing a total of 64,221 SNPs [58]. Among these variations, the majority consisted of intron variants (31,816), followed by missense (nsSNPs—14,231), coding sequence alterations (10,217), synonymous changes (5458), splice region variants (1069), frame shifts (652), stop gained (nonsense—275), in‐frame deletions (146), splice donor variants (103), splice acceptor variants (92), SNPs in the 5ʹ UTR region [59], SNPs in the 3ʹ UTR region [43], in‐frame insertions [40], start lost (nonsense—2) and stop lost (nonsense—1). The distribution highlights the prevalence of SNPs in intronic regions (49.30%), followed by missense variations (22.54%), coding sequences (15.97%), synonymous changes (8.5%), splice regions (1.43%), frameshifts (1%), nonsense mutations (0.44%), in‐frame deletions (0.23%), splice donor variants (0.16%), splice acceptor variants (0.15%), 5ʹ UTR region mutations (0.12%), 3ʹ UTR region mutations (0.07%) and in‐frame insertions (Tables S1–S4), (0.06%) (Figure 1A). Considering their types, quantity and potential impacts, the subsequent analysis focuses solely on the missense SNPs of MUC16 to investigate the influence of single nucleotide variants on its structural and functional characteristics.

### 3.2 Determination of Functional Impact of SNPs

The missense nsSNPs were meticulously filtered at each stage utilising diverse computational tools equipped with distinct algorithms. This rigorous process aimed to isolate the most deleterious mutations, potentially impacting the structure and functionality of MUC16. Subsequently, these detrimental mutations were singled out for comprehensive evaluation, while mutations predicted to be neutral by the tools were excluded from further consideration.

#### 3.2.1 Protein Sequence and Structure Homology‐Based Methods

The missense mutations sourced from the Ensembl dbSNP database underwent scrutiny via POLYPHEN‐2. Both the HumDiv and HumVar datasets, which are models trained within PolyPhen‐2, were employed for assessment. Among the total 14,231 SNPs obtained, the HumDiv dataset indicated 4017 substitutions as ‘probably damaging’, 3550 as ‘possibly damaging’ and classified 6653 as ‘benign’. Conversely, the HumVar model predicted 9098 mutations as ‘benign’, 2913 as ‘possibly damaging’ and 2208 as ‘probably damaging’. Notably, 2049 mutations were identified as ‘probably damaging’ by both the HumDiv and HumVar models, constituting the subset chosen for subsequent analysis (Figure 1B).

#### 3.2.2 Sequence Homology‐Based and Evolutionary Conservation Approaches

The missense SNPs identified as damaging by Polyphen‐2 underwent additional evaluation via sequence homology‐based methods, namely SIFT and PROVEAN. Out of the 2049 SNPs flagged as pathogenic by Polyphen‐2, SIFT predicted 1624 as ‘damaging’. Similarly, PROVEAN identified 667 missense SNPs as ‘deleterious’, all of which had also been identified as deleterious by SIFT. Remarkably, there were 567 mutations that were concurrently identified as detrimental by both tools (Figure 1B).

#### 3.2.3 Supervised Learning‐Based Approaches

Out of the 564 SNPs predicted as pathogenic by homology‐based tools, Snap2 identified 330 as pathogenic, while MutPred further refined this list to 84 SNPs predicted to have deleterious functional effects (Figure 1B and Table S1). Notably, all 84 of these pathogenic SNPs were located within the SEA domains of MUC16. MUC16 spans a total of 14,507 amino acids and contains 16 SEA domains, distributed as follows: SEA1 (12072–12193), SEA2 (12228–12349), SEA3 (12386–12507), SEA4 (12542–12663), SEA5 (12697–12818), SEA6 (12853–12974), SEA7 (13009–13130), SEA8 (13165–13286), SEA9 (13321–13442), SEA10 (13477–13598), SEA11 (13633–13754), SEA12 (13789–13909), SEA13 (13922–14043), SEA14 (14073–14193), SEA15 (14198–14309) and SEA16 (14319–14438).

Among the SEA domains, SEA5 (amino acids 12,697–12,818) was selected for detailed analysis due to its high sequence homology (> 80%) with other SEA domains [21], making it a suitable representative for exploring broader SEA domain characteristics. Among the 84 most deleterious SNPs identified within SEA domains, four SNPs: L12811P, Y12804N, C12771Y and D12768Y were selected for in‐depth study as they were all located specifically within the SEA5 domain. The selection of these SNPs was based on their strong pathogenic predictions across multiple computational tools and their presence in a highly conserved, representative domain, suggesting a high likelihood of functional and structural relevance.

### 3.3 Analysis of Protein Stability

The stability of the protein due to pathogenic mutations was assessed using the I‐mutant stability prediction tool. Among the selected substitutions, three SNPs (L151P, Y144N and C111Y) were predicted to decrease protein stability with respective DDG scores of −1.85, −1.76 and −0.13. However, the substitution D108Y was predicted to increase the protein's stability, with a DDG score of 0.51.

Furthermore, the stability analysis was further validated through performing another computational tool MUpro. MUpro predicted the stability effect of amino acid substitutions of *MUC16* protein based on single site mutations from sequences. Among the selected substitutions, four SNPs (L151P, Y144N, C111Y and D108Y) were predicted to decrease protein stability with respective delta G scores of −2.36, −1.46, −0.54 and −0.05. So, all of the scores indicated decreased stability.

### 3.4 Identification of the Domains in MUC16

The InterPro tool was instrumental in delineating the domain structure within the MUC16 protein and discerning the positions of nsSNPs across various domains. The protein consists of distinct regions: an N‐terminal domain housing a TR domain and a C‐terminal domain incorporating a cytoplasmic tail. Specifically, the TR domain in MUC16 comprises approximately 60 repeats of 156 amino acids, accompanied by 16 homologous SEA (Sea urchin sperm protein, Enterokinase and Agrin) domains interspersed with proline, serine and threonine‐rich sequences. Notably, among these, SEA1‐SEA12, positioned closer to the N‐terminal, exhibit substantial sequence homology (> 70% sequence identity), while SEA13‐SEA16, situated proximal to the membrane, display a relatively lower sequence identity (approximately 20%–64%) (Figure S2A).

Structurally, the SEA domain adheres to a canonical pattern, manifesting a Ferredoxin‐like fold characterised by an α/β sandwich structure (Figure S2B). This structure features two anti‐parallel β‐sheets, with the first sheet comprised of four strands, including three long β‐strands and a shorter one. Distinctive anti‐parallel β‐bulges introduce a twist in the third strand of the sheet. The second β‐sheet consists of two very short strands, each forming a β‐hairpin, and the structure also includes several α‐helices of varying lengths (Figure S2B).

Within the SEA5 domain, three cysteine residues are present, with Cys58 uniquely located in a solvent‐exposed loop. The remaining Cys91 and Cys11 are conserved among all 16 human MUC16 SEA domains and form a disulfide bond linking strands β2 and β3 (Figure S2B). Additionally, the selected nsSNPs (L12811P, Y12804N, C12771Y and D12768Y) are positioned in the catalytic kinase domain (Figure S2A).

### 3.5 Selection of Deleterious nsSNPs of MUC16 and Corresponding Conservation Analysis

**FIGURE 2:**
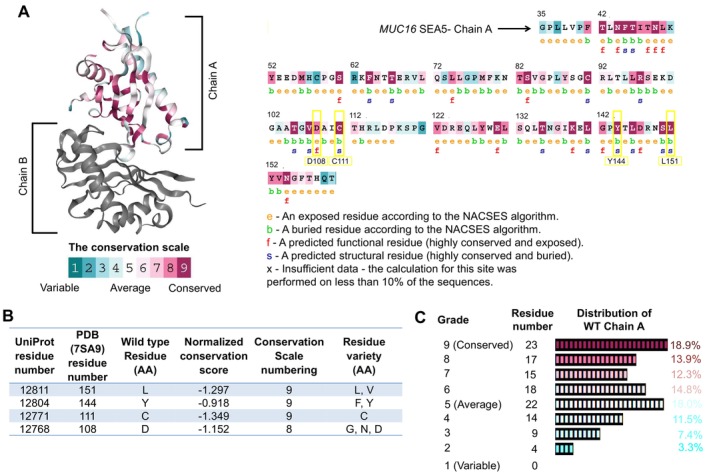
(A) ConSurf analysis of human MUC16 SEA5 domain‐Chain A residues. Final four predicted pathogenic residues (D108Y, C111Y, Y144N and L151P) are marked with a yellow box. (B) Conservation analysis of WT amino acids at the position of deleterious mutations. (C) Distribution residue number according to conservation grade scale by ConSurf analysis.

The investigation into the biology of MUC16 structure, particularly within the SEA5 domain (Figure S2A), unveiled a high degree of amino acid conservation across generations. Approximately 69.6% of the amino acid residues within the SEA5 domain displayed conservation scores above the average (> 5), with 18.9% of these residues showcasing the highest conservation scores. Notably, the four most pathogenic SNPs L12811P, Y12804N, C12771Y and D12768Y (PDB ID:7SA9_A, corresponding to residue numbers L151P, Y144N, C111Y and D108Y) were found to be situated within this conserved region (Figure 2A–C). This substantiates the notion that these specific SNPs exert a discernible impact on the structure and function of the protein, further corroborating their potential significance within this conserved domain. Moreover, the MEGA 11 package was facilitated to create a phylogenetic tree. The phylogenetic tree analysis exposed that MUC16 SEA5 domain and *Callorhinus ursinus* closed neighbours (Figure S3).

### 3.6 Modelling and Structural Validation Analysis

**FIGURE 3:**
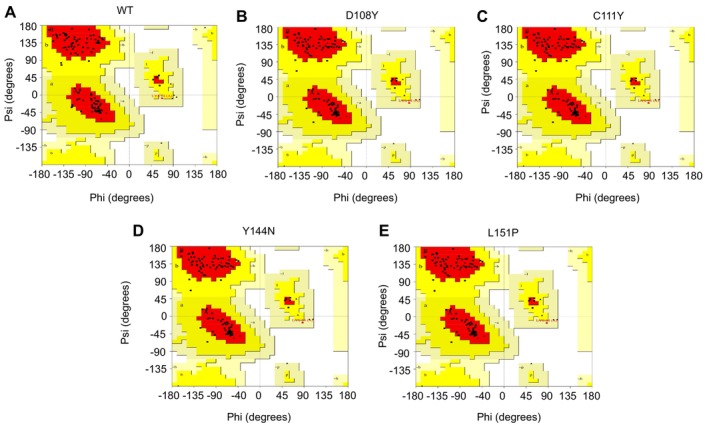
Structural validation MUC16 SEA5 domain WT (A) and mutant proteins (B–E) by Ramachandran plot. Here, in the figure red colour is the favoured region, yellow colour is the additionally allowed region, cream colour is the generously allowed region and the white colour is the disallowed region.

The quality assessment of the modelled crystal structures for both the WT and mutant proteins was determined by generating Ramachandran plots using the PROCHECK server. For the WT protein, the plot indicated 98 residues (92.5%) within the favoured region, 7 residues (6.6%) in the additionally allowed region and merely 1 residue (0.9%) within the generously allowed region (Figure 3A and Table S1). Meanwhile, the mutant variants D108Y, C111Y and Y144N exhibited 97 residues (91.5%) in the favoured region, 8 residues (7.5%) in the additionally allowed region, and again, only 1 residue (0.9%) in the generously allowed region (Figure 3B–D and Table S1). However, in the case of the L151P mutant protein, the Ramachandran plot displayed 96 residues (91.4%) within the favoured region, 8 residues (7.5%) within the additionally allowed region, and again, only 1 residue (0.9%) within the generously allowed region (Figure 3E and Table S1). Regarding the residue composition, the WT and mutant proteins D108Y, C111Y and Y144N exhibited a total of 105 non‐glycine and non‐proline residues, 1 end residue (excluding glycine and proline), 11 glycine residues and 0 proline residues. Interestingly, the mutant variant L151P displayed a similar number of residues, differing only by the presence of an additional proline residue.

### 3.7 Development of Mutant MUC16 Protein

**FIGURE 4:**
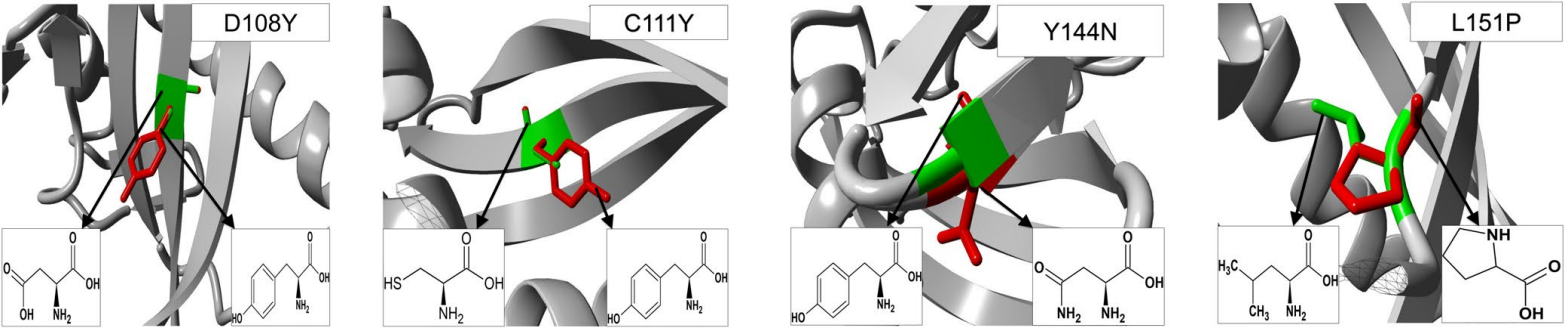
Superimposed structures of MUC16 SEA5 domain WT (green colour) and mutant (red colour) models to visualise the stereochemical conformation of wild type and mutant residues at D108Y, C111Y, Y144N and L151P positions.

To develop a mutant version of protein structure, we substituted specific amino acids in MUC16 which are shown in Figure 4. Variant D108Y and C111Y were substituted by a larger amino acid, tyrosine (181.2 g/mol). This larger tyrosine can disrupt interfaces where proteins interact with each other, affecting signalling pathways, structural integrity or complex formation. On the other hand, the mutant variant L151P results in proline (115.1 g/mol) in place of leucine (131.2 g/mol) and the mutant variant Y144N replaces tyrosine (181.2 g/mol) with small‐scale asparagine (131.1 g/mol). These two substitutions can create an empty space in the core of the SEA5 domain of *MUC16* and can also affect the transduction between other SEA domains. Moreover, these substitutions may lead to protein folding, contributing to disease. Furthermore, mesothelin, acting as a *MUC16* functional partner in cancer development, may become more prone to bind with these mutant variants [48].

### 3.8 WT and Mutant Proteins Simulation

**FIGURE 5:**
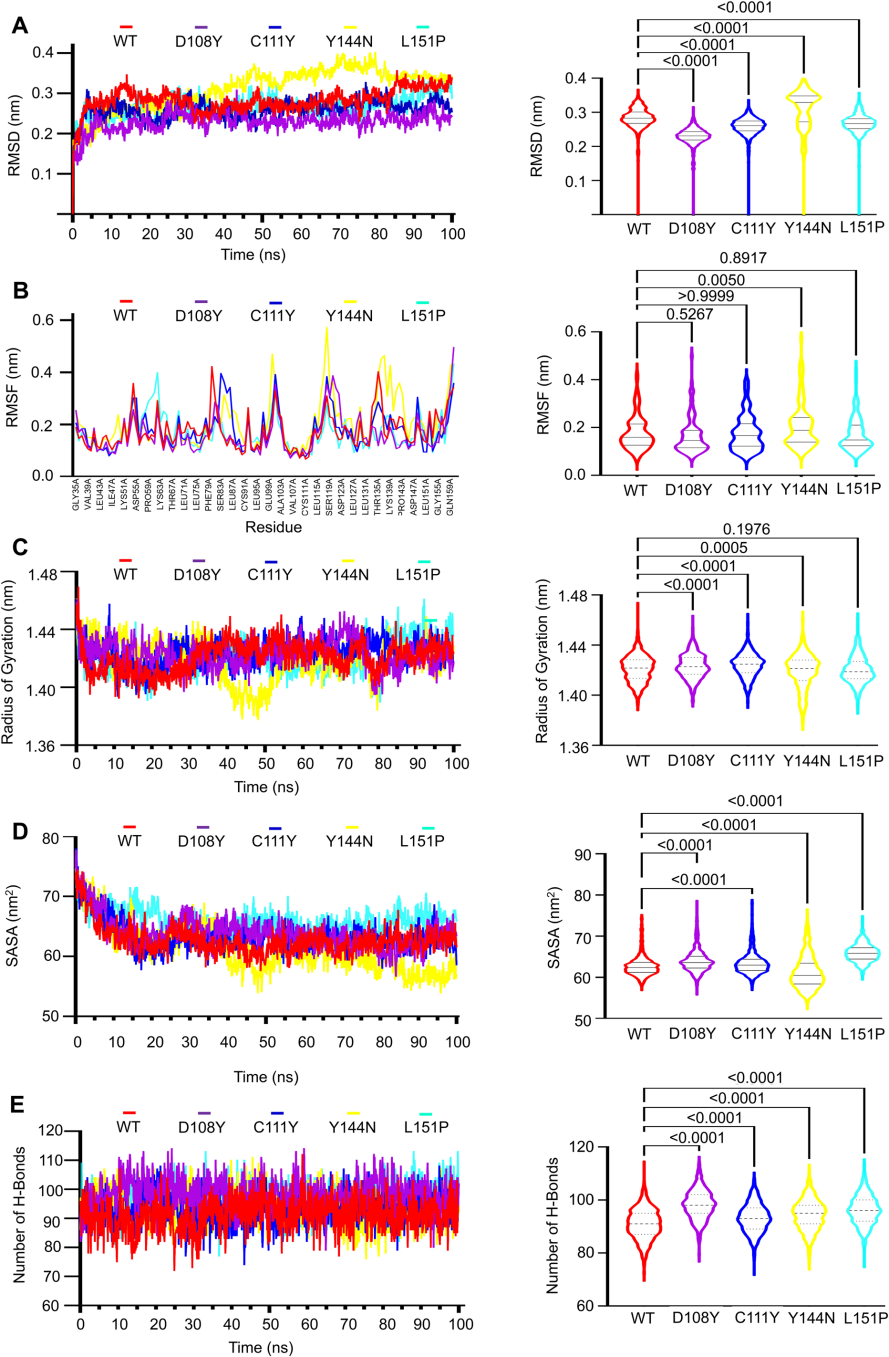
(A) C‐α atoms depicts the RMSD values of WT and mutant proteins, (B) Residue‐wise RMSF values of WT and mutant proteins, where *x* coordinate is the sequences of amino acid residue, (C) Calculation of radius of gyration (Rg), after determining the centre of mass of the solute, (D) Solvent Accessible Surface Areas (SASA), consists of all the points that the centre of the water probe [i.e., the nucleus of the oxygen atom in the water molecule] can reach while rolling over the solute, and (E) Number of hydrogen bonds of WT and mutant proteins. (C–G, right column) The RMSD, RMSF, Rg, SASA and H‐bond frequency distributions for the WT and four chosen mutants (D108Y, C111Y, Y144N and L151P) are shown by violin plots. The highest, lowest and densest points in the data are shown by the top, bottom and breadth of the violin, respectively. The continuous and dotted lines represent, respectively, the median and quartiles of the data distribution Here, the symbol coding scheme in the illustration describes: WT (red colour), D108Y (purple colour), C111Y (blue colour), Y144N (yellow colour), L151P (cyan colour) and violins plots indicates statistically significant difference (*p* < 0.0001).

Molecular dynamics simulations were performed over 100 ns to scrutinise the structural alterations in the MUC16 SEA5 domain, comparing the WT and mutant proteins in conditions mirroring the human physiological system. Using the trajectory files of both the wild type and four selected detrimental mutants, the RMSD was computed to ascertain the conformational stability of the proteins throughout the simulation. Throughout the simulation period, all of the Y114N variants had greatly diverged from the wild type, according to RMSD analysis (Figure 5A). WT and the mutants D108Y, C111Y, Y144N and L151P have average RMSD values of 0.28, 0.22, 0.26, 0.31 and 0.27 nm, respectively. The variation was greatest for Y144N, which peaked at 0.29 nm at 30 ns and plateaued at 0.34 nm at 83.2 ns. With increases at 14 and 85 ns and dips at 32 and 72 ns, WT displayed larger variances. The profiles of C111Y and L151P were comparable, with a few minor variations of about 20 ns. D108Y's RMSD values remained lower and more consistent throughout the course of 100 ns.

The root‐mean‐square fluctuation (RMSF) of every amino acid residue was determined using the MD trajectories in order to comprehend the dynamic consequences of specific detrimental mutants on the MUC16 protein (Figure 5B). Both the D108Y and C111Y mutants display similar fluctuations in residues positioned at 53–66, 79–97, 97–105 and 114–125; however, the C111Y mutant exhibits a greater fluctuation tail. Moreover, the WT and mutant Y144N show fluctuations in residues ranging from 55 to 56, 62–63, 79–92, 98–105, 114–125 and 132–145, with higher fluctuation values compared to the D108Y and C111Y mutants. In a comparative analysis, the mutant Y144N displays a greater tail in residue fluctuations, reaching values of 0.47 and 0.57 nm. Conversely, the mutant L151P exhibits fluctuations at residue positions 57–64, 98–104 and 147–149, albeit without a significant increase in value.

The Rg (R adius of gyration) values were computed for every trajectory to evaluate the overall form of the protein structure resulting from the substitution of amino acids. Mutants had greater Rg values than WT up to 36 ns, according to the Rg analysis (Figure 5C). After peaking at 9.7 and 25.5 nm, Y144N fell below 1.38 nm in the 37–53 nm range and also produced the greatest flexibility while requiring minimal compactness. Between 76 and 82 ns, Rg dropped to 1.38 nm, and at 92.7 ns, it increased. At 1.43 nm, C111Y was stable. With dips at 28.5 ns (1.39 nm) and 75.7–83 ns, D108Y averaged 1.43 nm and peaked at 1.47 nm at 71 ns. 1.40 nm for 32 ns, followed by 1.43 nm was WT. For 82 ns, L151P was 1.43 nm and after that, 1.44 nm.

The average SASA values for mutants D108Y, C111Y and L151P were 63.911, 63.394 and 65.902 nm^2^, respectively, indicating higher expansion compared to WT (62.7 nm^2^) conformation (Figure 5D). The Y144N mutant had a lower average of 61.243 nm^2^ but showed deviations at 10 ns (65 nm^2^), 20 ns (58.6 nm^2^), 38 ns (64.8 nm^2^), 40 ns (59.8 nm^2^), 48.9 ns (53.4 nm^2^), 56 ns (64.79 nm^2^) and 95.9 ns (53.9 nm^2^) may result from the substitution of one amino acid, changing the size of the protein surface and other properties [60].

The calculation of hydrogen bonds within the proteins indicated that the total number of hydrogen bonds in all the mutants closely resembled that of the WT variant 7SA9 (Figure 5E). However, notably, during the simulation of the mutant protein Y144N, there were significantly larger deviations observed in the number of hydrogen bonds compared to both other mutant proteins and the WT variant.

### 3.9 WT and Mutant Proteins Docking Analysis With N‐Linked‐Glycan (NLG) and O‐Linked‐Glycan (OLG)

**FIGURE 6:**
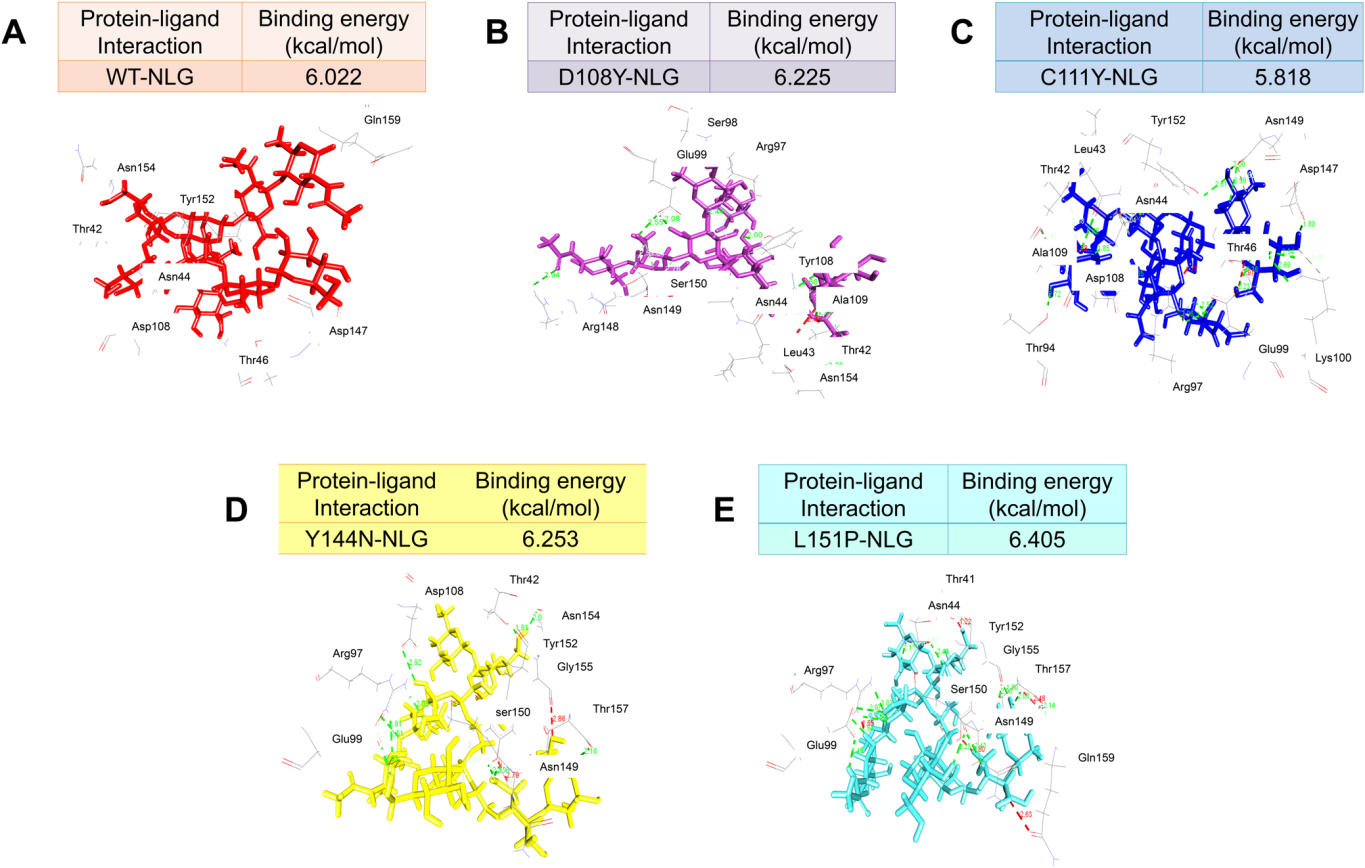
Interacting amino acid residue between MUC16 SEA5 domain WT (A) and mutant proteins (B–E) with NLG.

The comparison between molecular docking of WT and mutant variants revealed variations in their binding energy. The WT‐NLG complex exhibited a lower binding energy compared to all mutant protein‐NLG complexes, except for the C111Y‐NLG complex (Figure 6A–E). In the WT protein, conventional hydrogen bonds (CHs) were observed in Asn44, Tyr152, Gln159, Thr46, Asp108, Asp147 and Asn154. However, the mutant variants displayed CHs in residues Thr42, Arg97, Tyr108, Arg148, Asn149, Glu99, Ala109, Ser98, Thr94, Tyr152, Thr157, Gly155 and Ser150, which were not present in the WT (Table S2). The comparison of NLG complexes between WT and mutant variants revealed differences in their binding poses, as depicted in Figure S4A. Additionally, the docking of OLG against the WT showed lower binding energy compared to all mutant variants, as illustrated in Figure S5A–E. In the WT, Cs‐type interacting residues included Asn44, Thr46, Arg97 and Tyr152. However, CHs‐type interacting residues observed in the mutants but absent in the WT were Asn149, Ser98, Glu99 and Lys100 (Table S3). A superimposition was carried out (Figure S5F) to assess the deviation of OLG when docked with both WT and mutant proteins. This comparison highlighted noticeable differences in the positioning of the bound NLG and OLG ligands between the WT and mutant protein complexes. Specifically, higher deviations were observed in the D108Y and C111Y mutant protein complexes, as depicted in Figures S4 and S5F.

### 3.10 WT and Mutant Proteins‐N‐Linked‐Glycan (NLG) Docking Complexes Simulation

**FIGURE 7:**
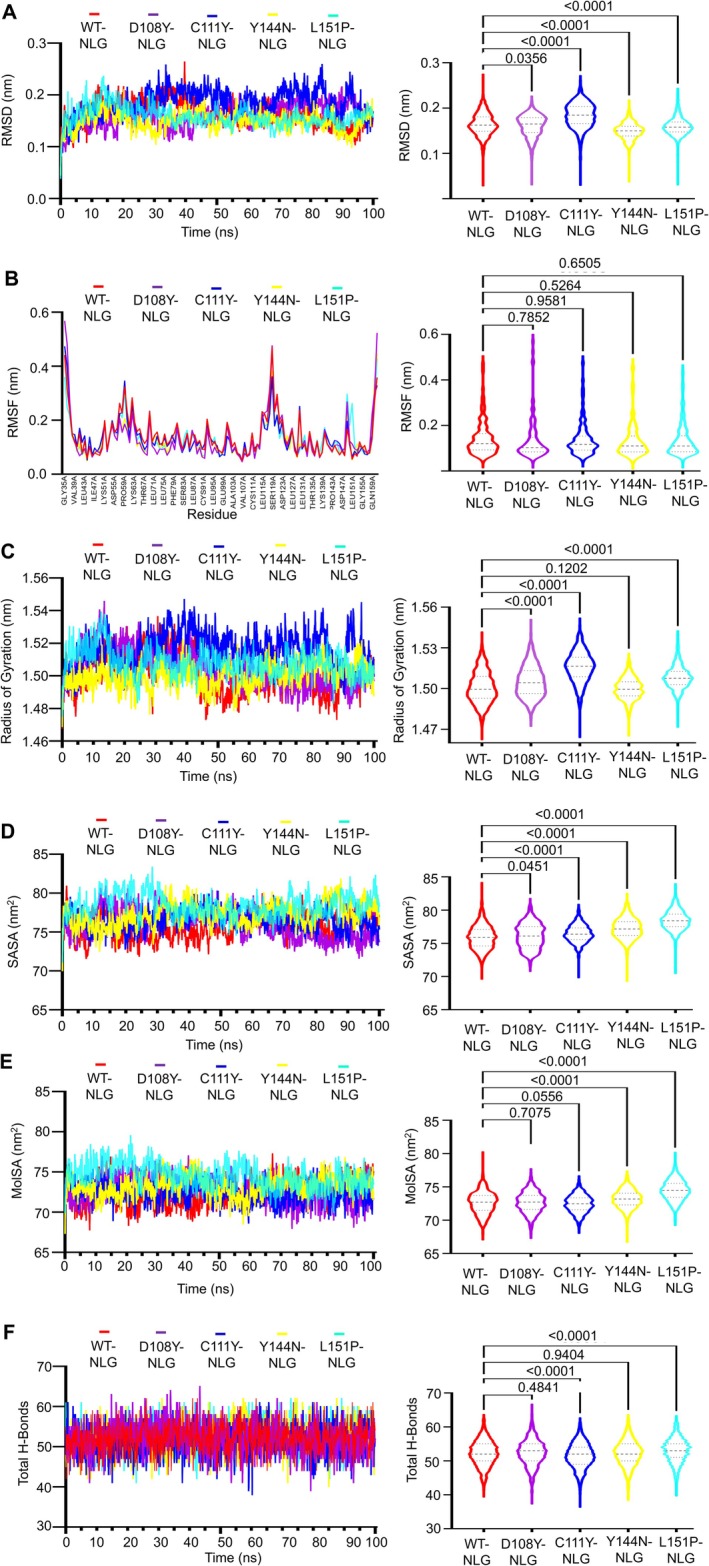
(A) RMSD values the of C‐α atoms of WT and mutant proteins of MUC16 SEA5 domain docked complexes with NLG. (B) Residue‐wise RMSF values of WT and mutant proteins docked complexes with NLG, where x‐axis coordinate is the sequences of amino acid residues, (C) Calculation of radius of gyration (Rg), after determining the centre of mass of the solute. (D) Solvent Accessible Surface Areas (SASA), consists of all the points that the centre of the water probe (i.e., the nucleus of the oxygen atom in the water molecule) can reach while rolling over the solute. (E) Molar Surface Area (MolSA), is the Van der Waals surface from the viewpoint of a solvent molecule and (F) Total hydrogen bonds of WT and mutant proteins after docked with NLG complexes. (B–G, right column) The RMSD, RMSF, Rg, SASA, MolSA and total H‐bond frequency distributions for the WT and four chosen mutants (D108Y‐NLG, C111Y‐NLG, Y144N‐NLG and L151P‐NLG) are shown by violin plots. The highest, lowest and densest points in the data are shown by the top, bottom and breadth of the violin, respectively. The continuous and dotted lines represent, respectively, the median and quartiles of the data distribution. Here, the symbol coding scheme in the illustration describes: WT‐NLG (red colour), D108Y‐NLG (purple colour), C111Y‐NLG (blue colour), Y144N‐NLG (yellow colour), L151P‐NLG (cyan colour) and violins plots indicates statistically significant difference (*p* < 0.0001).

The study aimed to assess how mutations in the MUC16 SEA5 domain impact glycosylation patterns, focusing on the interaction between N‐linked glycan (NLG) and mutant proteins. Analysis of RMSD revealed varying behaviours among NLG complexes with mutants. Y144N‐NLG showed higher average RMSD (0.194 nm) compared to WT‐NLG and other mutants (0.161–0.183 nm). Both Y144N‐NLG and L151P‐NLG initially increased RMSD followed by decreases, but only Y144N‐NLG stabilised. WT‐NLG and certain mutants maintained consistent values for about 30 ns before exhibiting fluctuations. C111Y‐NLG displayed decreased RMSD after 54.5 ns, while WT‐NLG increased, and D12868Y‐NLG decreased until ~45 ns, stabilising thereafter (Figure 7A).

The Y144N‐NLG mutant protein displayed fluctuations in residues 51–54, 113–125 and 148–150, showing relatively lower residual fluctuation and a lesser tail‐up pattern compared to WT‐NLG, D108Y‐NLG, C111Y‐NLG and L151P‐NLG mutants. In contrast, both WT‐NLG and D108Y‐NLG, C111Y‐NLG and L151P‐NLG mutants exhibited significant fluctuation from residue numbers 55–63, 68–70, 113–124 and 147–150. Additionally, fluctuations were observed from 129 to 130 in WT‐NLG and C111Y‐NLG mutants, with the L151P‐NLG mutant showing a more pronounced tailing‐up pattern in this region. The presence of N‐ and C‐terminal domains likely contributes to most of the variations observed at the protein's beginning and end (Figure 7B).

The average Rg values for WT‐NLG, D108Y‐NLG, C111Y‐NLG, Y144N‐NLG and L151P‐NLG mutants were measured at 1.5, 1.49, 1.52, 1.49 and 1.51 nm, respectively. Throughout the 100 ns simulation, the Y144N‐NLG mutant displayed no significant variance and remained stabilised for the entire duration. The average Rg value of the D108Y‐NLG mutant (1.49 nm) was slightly lower than that of the WT‐NLG (1.5 nm), while the Rg values for the C111Y‐NLG (1.52 nm) and L151P‐NLG (1.51 nm) mutants were higher than that of the WT NLG. Following the initial 42.5 ns, the Rg value of the D108Y‐NLG mutant gradually decreased, while the WT‐NLG value sharply declined during the same period. Conversely, the Rg value of the mutant protein C111Y‐NLG deviated notably at 6, 27.4 and 90 ns, and the mutant L151P‐NLG exhibited significant deviation from 0 to 29 ns, as depicted in Figure 7C.

The Y144N‐NLG mutant maintained a consistent SASA value but experienced fluctuations at 31.4 and 81.5 ns, averaging at 77.22 nm^2^. Mutant C111Y‐NLG showed increased SASA values at various points, averaging at 76.43 nm^2^, higher than WT‐NLG. D108Y‐NLG had minor fluctuations, averaging at 76.09 nm^2^. WT‐NLG initially surged, then stabilised at 75.68 nm^2^. Mutant L151P‐NLG had a higher average SASA (74.50 nm^2^) than all, with variations between 12.5 and 56 ns (Figure 7D).

Mutant L151P‐NLG had a greater average MolSA value (74.50 nm^2^) compared to WT‐NLG (72.45 nm^2^) and others, with fluctuations at specific points. WT‐NLG showed deviations at intervals, while D108Y‐NLG and C111Y‐NLG had minor variations. Y144N‐NLG maintained stability despite deviations during the 100 ns simulation (Figure 7E).

The average total number of hydrogen bonds for the WT was 52.14, which was lower compared to the mutant variants: D108Y‐NLG (52.4), Y144N‐NLG (52.3) and L151P‐NLG (52.99). Conversely, it was higher than that of the mutant protein C111Y‐NLG (51.50) as depicted in Figure 7F. Higher hydrogen bond numbers typically indicate stronger binding strength with NLG.

### 3.11 Protein–Protein Interaction of Mesothelin (PDB ID: 8CX3) With WT and Mutant Proteins

**FIGURE 8:**
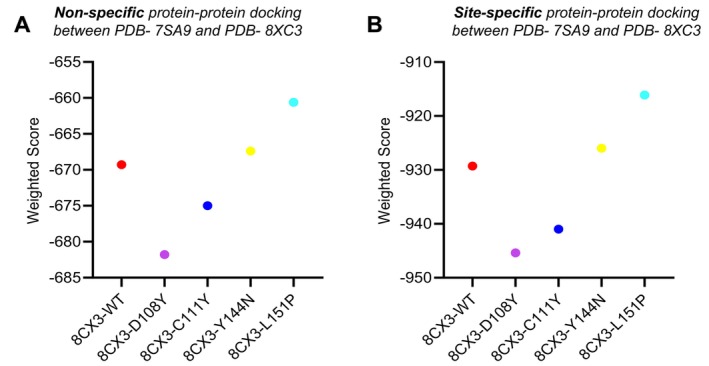
(A) (Non‐specific), and (B) (site‐specific) protein–protein docking weighted score of mesothelin (PDB ID: 8CX3) with MUC16 SEA5 domain WT and mutant proteins, WT and mutant protein complexes with NLG, and WT and mutant protein complexes with OLG.

**FIGURE 9:**
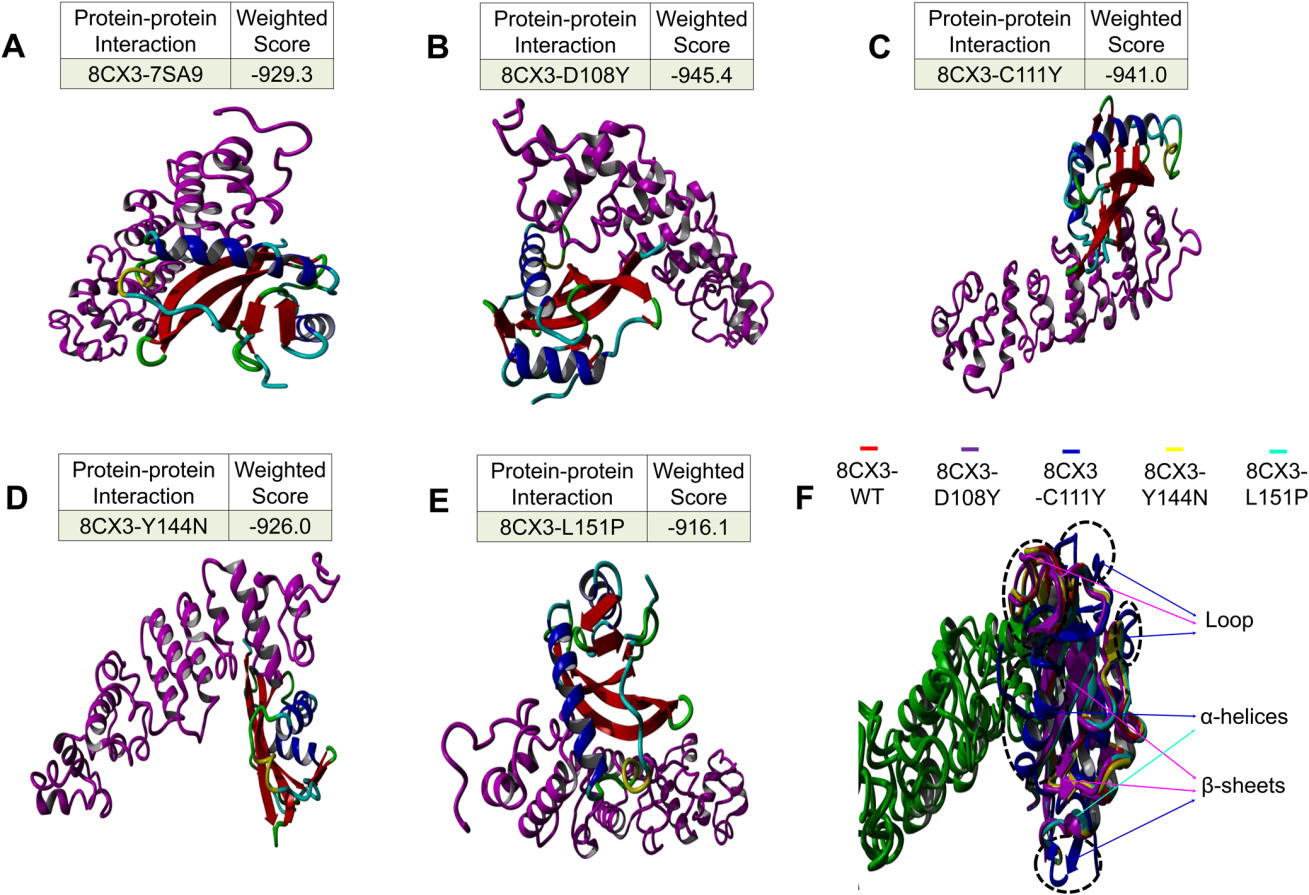
(A–E) Protein–protein docking of mesothelin (PDB ID: 8CX3) with MUC16 SEA5 domain WT and mutant proteins. (F) Superimposition of MUC16 SEA5 domain protein–protein docking complexes of mesothelin (PDB ID: 8CX3) with WT and mutant proteins.

Site‐specific docking in mesothelin with WT and mutant proteins showed lower weighted scores than non‐specific docking, consistently exceeding −900 weighted scores (Figure 8A,B). Site‐specific docking enables more compact binding to mesothelin, which is further understood by seeing the salt bond (SB) between mesothelin and WT and variants non‐bond interaction (Table S4). Upon superimposition, D108Y and C111Y mutants exhibited more deviation and notable changes in structural elements like α helices, β‐sheets and the β3‐α2 loop, unlike Y144N and L151P mutants (Figure 9F).

### 3.12 Mesothelin (PDB ID: 8CX3)‐WT and Mutant Proteins Simulation

**FIGURE 10:**
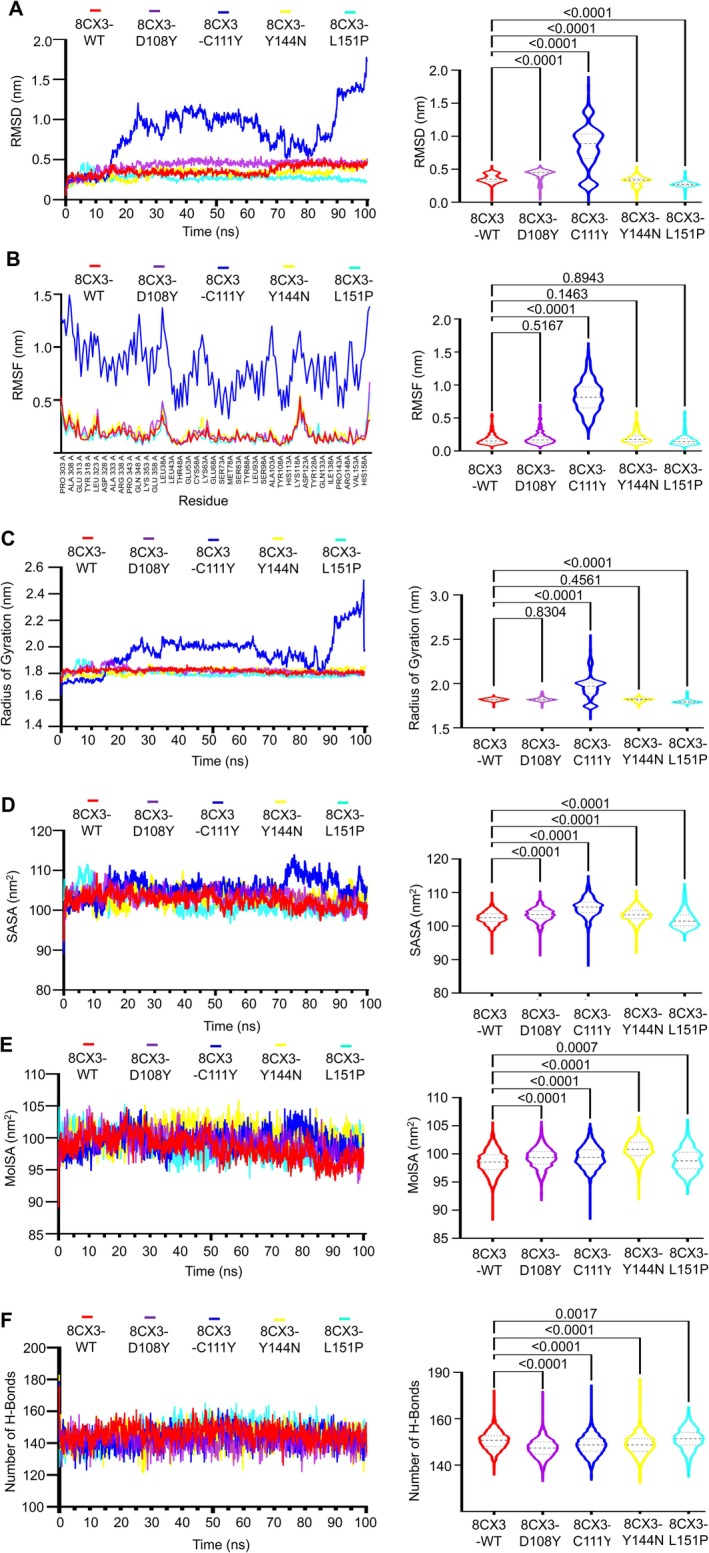
(A) RMSD values of the C‐α atoms of WT and mutant proteins docked complexes with mesothelin, (B) Residue‐wise RMSF values of WT and mutant proteins docked complexes with mesothelin, where x coordinate is the sequences of amino acid residue, (C) Calculation of radius of gyration (Rg), after determining the centre of mass of the solute, (D) Solvent Accessible Surface Areas (SASA), consists of all the points that the centre of the water probe (i.e., the nucleus of the oxygen atom in the water molecule) can reach while rolling over the solute, (E) Molar Surface Area (MolSA), is the Van der Waals surface from the viewpoint of a solvent molecule and (F) Number of hydrogen bonds of WT and mutant proteins after docking with mesothelin. (B–G, right column) The RMSD, RMSF, Rg, SASA, MolSA and H‐bond frequency distributions for the WT and four chosen mutants (8CX3‐D108Y, 8CX3‐C111Y, 8CX3‐Y144N and 8CX3‐L151P) are shown by violin plots. The highest, lowest and densest points in the data are shown by the top, bottom and breadth of the violin, respectively. The continuous and dotted lines represent, respectively, the median and quartiles of the data distribution Here, the symbol coding scheme: 8CX3‐WT (red colour), 8CX3‐D108Y (purple colour), 8CX3‐C111Y (blue colour), 8CX3‐Y144N (yellow colour), 8CX3‐L151P (cyan colour) and violins plots indicates statistically significant difference (*p* < 0.0001).

8CX3‐WT initially increased RMSD to 0.4365 nm, stabilised, and rose again to 0.45 nm around 67 ns. Mutant 8CX3‐D108Y peaked at 0.473 nm in the first 40 ns, stabilising at 0.46 nm thereafter (Figure 10A). Mutant 8CX3‐C111Y had an average RMSD of 0.84 nm with consistent large deviations. 8CX3‐Y144N averaged 0.324 nm, with slight increases at 25 and 88.2 ns. 8CX3‐L151P averaged 0.273 nm, with sharp deviations at 3–11 ns and moderate ones at 30 ns and 90 ns.

RMSF analysis highlighted significant fluctuations in specific residue ranges (302–308, 315–323, 35–41, 62–64, 69–70, 100–104, 114–124 and 148–153) for 8CX3‐WT, 8CX3‐D108Y, 8CX3‐Y144N and 8CX3‐L151P mutants (Figure 10B). Mutant 8CX3‐Y144N showed additional fluctuation in residues 319–320 and 58–60, while 8CX3‐D108Y displayed fluctuation in residues 353–358, and 8CX3‐L151P in residues 129–130. Remarkably, mutant 8CX3‐C111Y exhibited higher fluctuation, tailing up and deviation compared to WT and other mutants.

Average Rg was lower for 8CX3‐Y144N and 8CX3‐L151P mutants (1.8 nm) compared to 8CX3‐WT (1.818 nm), 8CX3‐D108Y (1.82 nm) and 8CX3‐C111Y (1.96 nm). 8CX3‐L151P showed increased Rg from 3.6 to 11.7 ns and then decreased after 35 ns, while 8CX3‐Y144N had a slight increase after 28 ns but remained stable. 8CX3‐D108Y displayed significant deviations from 6 to 24 ns, while 8CX3‐WT showed minor changes at 42 and 74.3 ns before stabilising. In contrast, 8CX3‐C111Y exhibited consistent large deviations and fluctuations throughout the simulation (Figure 10C).

The average SASA values were 102.94 nm (8CX3‐WT), 103.33 nm (8CX3‐D108Y), 105.62 nm (8CX3‐C111Y), 103.30 nm (8CX3‐Y144N) and 101.94 nm (8CX3‐L151P) (Figure 10D). Notably, 8CX3‐C111Y showed higher values and significant deviations at 13.6, 27, 73, 85, 91 and 97 ns. 8CX3‐Y144N, the second‐highest value, exhibited variance only at 23–36 ns, remaining stable otherwise. 8CX3‐WT and 8CX3‐D108Y remained relatively steady, with slight decreases at specific time points (8CX3‐WT at 52 ns and 8CX3‐D108Y at 12 and 24 ns). Conversely, 8CX3‐L151P displayed varied values at 5–10, 15, 35, 53 and 72 ns.

Mutant 8CX3‐Y12804 had a higher average MolSA (100.78 nm^2^) than 8CX3‐WT (98.53 nm^2^), 8CX3‐D108Y (99.30 nm^2^), 8CX3‐C111Y (99.45 nm^2^) and 8CX3‐L151P (98.85 nm^2^) mutants, stabilising after an increase at 22 ns (Figure 10E). 8CX3‐WT fluctuated between 20 and 35 ns and decreased after 80 ns. 8CX3‐D108Y remained stable for 86 ns, then showed a slight decrease. 8CX3‐C111Y initially increased for 26 ns, stabilised, and deviated after 73 ns. Conversely, 8CX3‐L151P displayed fluctuations at 10, 21, 36, 57 and 62 ns.

The number of H‐bonds analysis showed a higher count within mutant 8CX3‐L151P (146.74) compared to 8CX3‐WT (145.78), 8CX3‐D108Y (140.899), 8CX3‐C111Y (142.729) and 8CX3‐Y144N (143.034) (Figure 10F).

### 3.13 Principal Component Analysis Results (PCA)

**FIGURE 11:**
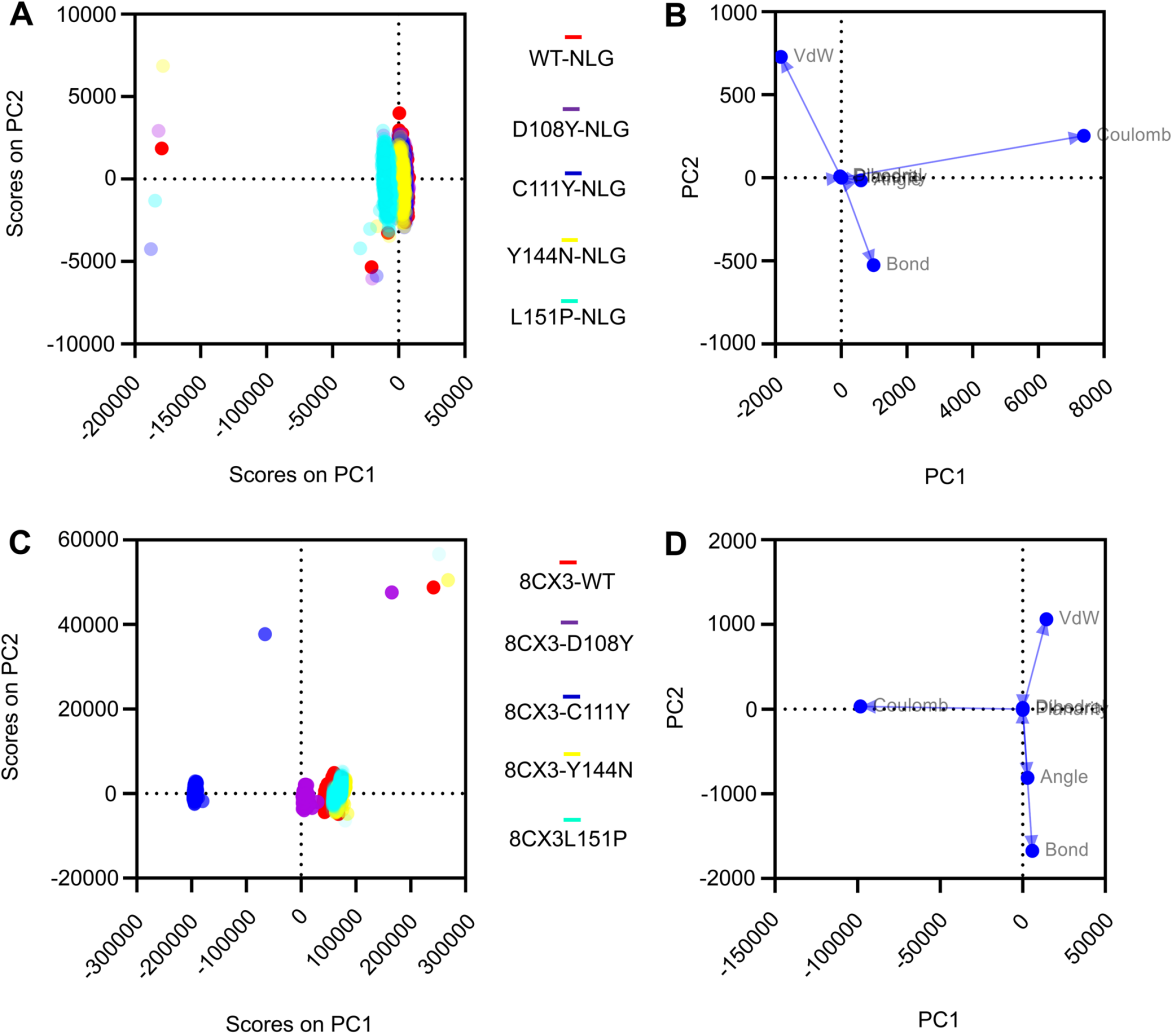
(A) Five data clusters were displayed within the score's plots, with each circle representing a single time point and displayed in a distinct colour. Here, the symbol coding scheme in the illustration describes: WT‐NLG (red colour), D108Y‐NLG (purple colour), C111Y‐NLG (blue colour), Y144N‐NLG (yellow colour) and L151P‐NLG (cyan colour). (B) Plotting loading points of NLG complexes using PCA of the structural and energy data. (C) Five data clusters were displayed within the score's plots, with each circle representing a single time point and displayed in a distinct colour. Here, the symbol coding scheme: 8CX3‐WT (red colour), 8CX3‐D108Y (purple colour), 8CX3‐C111Y (blue colour), 8CX3‐Y144N (yellow colour) and 8CX3‐L151P (cyan colour). (D) Plotting loading points of mesothelin (8CX3) complexes using PCA of the structural and energy data.

Energy and structural information of the WT and mutant protein complexes with NLG and mesothelin were visualised utilising PCA to get deeper clarification about the structural quality of all protein complexes during MD simulation. The PCA analysis of WT and mutant variants demonstrated the similarities and dissimilarities while all the complexes were in the simulation environment (Figure 11A). The score plot showed that all the mutant proteins exhibited a more clustered nature than WT‐NLG complexes, especially the Y144N‐NLG complexes. Moreover, except for D108Y‐NLG and L151P‐NLG complexes, all the mutant proteins overlapped with the WT‐NLG complex. The composition of bond energies, bond angle energies, dihedral angle energies, planarity energies, VdW energies and electrostatic energies makes up the energy and structural information [54]. The loading plot in Figure 11B described that Bond, Coulomb and VdW had a strong effect on protein stability. Furthermore, the mesothelin complexes cluster nature was higher in 8CX3‐L151P and 8CX3‐Y144N mutant proteins than in the 8CX3‐WT protein (Figure 11C). Also, the distance between the 8CX3‐D108Y and 8CX3‐C111Y mutant protein complexes was far from 8CX3‐WT, whereas the 8CX3‐L151P and 8CX3‐Y144N mutant protein complexes almost overlapped. The loading point in Figure 11D denoted that angle and bond had a negative impact on PC2, and Coulomb had a negative effect on PC1 (Table 1).

**TABLE 1:** The most deleterious amino acid substitutions predicted by the functional analysis tools.

Mutation	*MUC16* domains	POLYPHEN‐2	SIFT	PROVEAN	SNAP‐2	MUTPRED2
L14501R		PD	D	D	E	D
Y14487N		PD	D	D	E	D
L14464P		PD	D	D	E	D
L14457H		PD	D	D	E	D
W14453R		PD	D	D	E	D
F14452S		PD	D	D	E	D
L14450P		PD	D	D	E	D
S14437F	SEA16	PD	D	D	E	D
Y14436C	SEA16	PD	D	D	E	D
Y14436H	SEA16	PD	D	D	E	D
D14434Y	SEA16	PD	D	D	E	D
L14426R	SEA16	PD	D	D	E	D
G14418D	SEA16	PD	D	D	E	D
Y14408C	SEA16	PD	D	D	E	D
S14391F	SEA16	PD	D	D	E	D
D14390A	SEA16	PD	D	D	E	D
D14390Y	SEA16	PD	D	D	E	D
C14373Y	SEA16	PD	D	D	E	D
C14373G	SEA16	PD	D	D	E	D
L14332P	SEA16	PD	D	D	E	D
T14296P	SEA15	PD	D	D	E	D
G14294C	SEA15	PD	D	D	E	D
L14286P	SAE15	PD	D	D	E	D
L14243P	SEA15	PD	D	D	E	D
L14237H	SEA15	PD	D	D	E	D
Y14179C	SEA14	PD	D	D	E	D
G14177V	SEA14	PD	D	D	E	D
G14177D	SEA14	PD	D	D	E	D
H14171P	SEA14	PD	D	D	E	D
L14166R	SEA14	PD	D	D	E	D
D14158A	SEA14	PD	D	D	E	D
C14146F	SEA14	PD	D	D	E	D
C14126R	SEA14	PD	D	D	E	D
Y14123C	SEA14	PD	D	D	E	D
S14084F	SEA14	PD	D	D	E	D
T14082A	SEA14	PD	D	D	E	D
L14079P	SEA14	PD	D	D	E	D
L14074R	SEA14	PD	D	D	E	D
Y14029S	SEA13	PD	D	D	E	D
G14027D	SEA13	PD	D	D	E	D
L14026P	SEA13	PD	D	D	E	D
T14020I	SEA13	PD	D	D	E	D
L14016R	SEA13	PD	D	D	E	D
L13994R	SEA13	PD	D	D	E	D
D13993Y	SEA13	PD	D	D	E	D
V13978G	SEA13	PD	D	D	E	D
C13976R	SEA13	PD	D	D	E	D
Y13973C	SEA13	PD	D	D	E	D
G13970C	SEA13	PD	D	D	E	D
L13969R	SEA13	PD	D	D	E	D
L13959P	SEA13	PD	D	D	E	D
D13940G	SEA13	PD	D	D	E	D
T13931P	SEA13	PD	D	D	E	D
L13928P	SEA13	PD	D	D	E	D
D13704Y	SEA11	PD	D	D	E	D
L13689R	SEA11	PD	D	D	E	D
C13687R	SEA11	PD	D	D	E	D
L13646P	SEA11	PD	D	D	E	D
C13551F	SEA10	PD	D	D	E	D
C13551S	SEA10	PD	D	D	E	D
C13551Y	SEA10	PD	D	D	E	D
Y13492N	SEA10	PD	D	D	E	D
L13279P	SEA8	PD	D	D	E	D
L13255P	SEA8	PD	D	D	E	D
C12907F	SEA6	PD	D	D	E	D
L12811P	SEA5	PD	D	D	E	D
Y12804N	SEA5	PD	D	D	E	D
C12771Y	SEA5	PD	D	D	E	D
D12768Y	SEA5	PD	D	D	E	D
D12496Y	SEA3	PD	D	D	E	D
L12471P	SEA3	PD	D	D	E	D
R12446S	SEA3	PD	D	D	E	D
Y12163C	SEA1	PD	D	D	E	D
R12149C	SEA1	PD	D	D	E	D
C12146Y	SEA1	PD	D	D	E	D
D12143Y	SEA1	PD	D	D	E	D
D12143H	SEA1	PD	D	D	E	D
N12116I	SEA1	PD	D	D	E	D
L14501R		PD	D	D	E	D
Y14487N		PD	D	D	E	D
L14464P		PD	D	D	E	D
L14457H		PD	D	D	E	D
W14453R		PD	D	D	E	D
F14452S		PD	D	D	E	D

Abbreviations: D, deleterious/damaging/disease; E, effect; PD, probably damaging.

## 4 Discussion

The advent of the first human genome sequence has propelled the advancement of disease genetics [61]. Single nucleotide polymorphisms (SNPs) account for over 90% of genetic variations in humans and occur roughly every 200–300 base pairs [62, 63]. Extrapolating this data to the entire genome suggests a potential 11–15 million SNPs for research [64, 65]. Large‐scale genome‐wide association analyses employing SNPs are instrumental in deciphering the genetic variations influencing human phenotypic diversity [66] SNPs serve as crucial genetic markers in biomedical research, aiding in the identification of common genetic variants and their impacts—both detrimental and neutral—on protein function associated with various disorders [67, 68].

The study of SNPs is crucial due to their several advantages [69] SNPs occurring in functional gene regions can induce variations in protein structure and expression, contributing to diseases or benign phenotypic changes [70]. They serve to identify genetic disparities underlying specific phenotypes that might be otherwise challenging to detect [71]. Additionally, SNPs offer insights into evolutionary history [72]. These variations can exist in coding, noncoding, or intergenic regions of the genome. While SNPs in non‐coding and intergenic regions might impact proteins, non‐synonymous SNPs (nsSNPs) in coding regions—associated with amino acid substitutions—often exert more significant effects on protein structure and function [73]. Studies suggest that over 50% of hereditary genetic disorders stem from high‐risk nsSNPs altering protein function [56].

Understanding the impact of amino acid substitutions on protein structure, stability and function is crucial, considering not all reported mutations are pathogenic [59, 74]. The vast number of SNPs in the NCBI database makes in silico bioinformatics tools a valuable and efficient approach to detecting disease‐associated SNPs with functional significance. These tools also enable a thorough exploration of an individual's susceptibility to certain diseases, offering a cost‐effective and time‐saving method [75, 76].

This study marks the first use of diverse contemporary in silico methods to explore the structural and functional effects of missense nsSNPs in MUC16. While various studies have implicated multiple mutations in different diseases [77, 78] the significance of MUC16 variants compared to natural polymorphisms remains poorly understood. MUC16 stands out as one of the most frequently mutated genes in various cancers [79], with its overexpression linked to metastasis and tumourigenesis in ovarian, breast, pancreatic and colorectal cancers [80]. Given its irregular overexpression across tumours, MUC16 holds promise as a prospective target for cancer diagnosis and therapy [81]. As nsSNPs notably impact protein structure and function [82], this study specifically identified and examined harmful nsSNPs in the MUC16 gene. Given the direct involvement of missense mutations in disease pathology, only missense variations were analysed in this investigation.

The study employed various computational techniques to filter missense nsSNPs, identifying 84 pathogenic mutations out of 14,231 nsSNPs in MUC16. Surprisingly, out of 84 pathogenic mutations, 71 were found in the SEA domain of MUC16. For further analysis of MUC16 SEA domains, the SEA5 domain is especially well‐suited because of a number of important considerations. First off, the glycosylation and antibody reactivity of the SEA5 domain have been previously described for several MUC16/CA125. Second, other SEA domains (SEA1–12) that are situated farther from the membrane share more than 80% homology with the SEA5 domain [21]. Because of its high degree of homology, SEA5 is a great representative model since it shows that knowledge obtained from studying it will probably also apply to these other domains. This makes it a good choice for in‐depth research projects that try to comprehend the overall structure–function correlations, glycosylation patterns and protein–protein interactions of MUC16 SEA domains.

Using tools like PROVEAN, SIFT, PolyPhen‐2, SNAP‐2, MutPred, I‐Mutant3.0 and MUpro four nsSNPs (L151P, Y144N, C111Y and D108Y) within the SEA5 domain of MUC16 were selected for amino acid conservation analysis. Amino acid conservation reflects the balance between its inherent mutational tendency and the need to maintain structural and functional integrity [83]. All four mutations exhibited high conservation scores [8, 9] across generations, indicating their likelihood of being pathogenic and impacting MUC16's structure and function. Moreover, the phylogenetic tree uncovers that the MUC16 SEA5 domain and *Callorhinus ursinus* (Northern fur seal) closely related each other. Furthermore, the stability analysis revealed that L151P, Y144N and C111Y substitutions decreased protein stability, while the D108Y substitution increased it. As the percentages Ramachandran plot of residues were in the acceptable ranged more 90% in the most favoured regions [40], the modelled structure of WT and mutant protein were regarded as validate. Moreover, no residues were found in the disallowed regions.

In a 100 ns molecular dynamics simulation, the MUC16 SEA5 domain's WT and mutant variants (D108Y, C111Y and Y144N) were evaluated. Mutants D108Y and C111Y demonstrated stability and minimal fluctuation in RMSD and RMSF values, while Y144N showed significant deviations. The mutants exhibited increased Rg values, indicating reduced compactness, with Y144N displaying more deviation, suggesting less compactness. Y144N also showed expanded SASA values, indicating reduced stability despite being higher than WT and other variants.

Glycans (compounds consisting of a large number of monosaccharides linked glycosidically) dictate proteolysis patterns and directly mediate ligand‐receptor interactions, oncogenic signalling transduction, immune recognition, migration and both cell–cell and cell‐matrix adhesion [84, 85, 86]. Oligosaccharides (shorter and a subset of glycans) can be covalently attached to the nitrogen atom of the amide group in the asparagine side chain, and to the oxygen atom of the hydroxyl group of serine (Ser) or threonine (Thr) residues in a protein [87]. As a result, we conducted non‐covalent interactions with NLGs and O‐linked glycan with MUC16 protein. Even if glycan attachment to proteins is covalent, it is still authentic and beneficial to research the non‐covalent interactions between the N‐glycan and O‐glycan with MUC16. Both glycans display a variety of non‐covalent interactions, such as hydrogen bonds, VdW forces and electrostatic interactions, with the MUC16 protein's surface and surrounding environment. Docking studies revealed significant alterations in the binding pocket for all mutants compared to WT, indicating a higher likelihood of malignancy due to changes in NLG and OLG binding associated with MUC16 metastasis characteristics [21]. Further MD simulations of variants‐NLG complexes showed Y144N‐NLG having lower RMSD and RMSF values, denoting better stability and fewer residue fluctuations than other WT and mutant variant complexes. Y144N exhibited lower Rg values, indicating higher compactness and tighter complex formation compared to all NLG complexes. Conversely, L151P showed higher SASA values and deviation. Notably, Y144N's higher SASA compared to WT, D108Y and C111Y suggests increased solvent exposure, enhancing NLG interaction accessibility. Varying hydrogen bond numbers suggested different binding strengths, with Y144N and L151P‐NLG mutants displaying higher values than WT.

In the interactions of mutant variants complexes, the formation of CHs and Cs revealed enhanced binding interactions. Protein–protein docking of mesothelin with WT and mutant MUC16 proteins, along with their NLG and OLG complexes, was conducted to assess potential contributions to tumour implantation and peritoneal spread via cell adhesion (Rump et al. 2004). A high weighted score (> 900) and mesothelin binding to the proteins indicated malignant properties. In a 100 ns molecular dynamics simulation of mesothelin complexes, 8CX3‐C111Y displayed notable RMSD fluctuations, whereas 8CX3‐Y144N and 8CX3‐L151P remained stable with lower RMSD values than WT. RMSF analysis revealed reduced residual flexibility in 8CX3‐L151P. Rg and SASA values indicated that 8CX3‐Y144N and 8CX3‐L151P mutants exhibited higher compactness and relatively elevated SASA values, indicating stable interactions with mesothelin across most of the simulation, suggesting improved binding potential.

The Y144N‐NLG complex exhibited greater stability compared to other NLG complexes according to the PCA analysis. This suggests that modifying the WT protein into the Y144N mutant variant could potentially enhance the glycosylation nature. Analysis of mesothelin simulation complexes indicated that the 8CX3‐L151P and 8CX3‐Y144N mutant proteins, when binding at the N‐terminus of cell surface mesothelin, might contribute more to cancer development than the 8CX3‐WT complex. Table 2 provides further hypotheses, suggesting that the L151P substitution could have a detrimental effect when linked to mesothelin. However, considering all other hypotheses derived from MD simulations regarding the impact of these substitutions on protein stability, it is notable that the Y144N substitution displayed the highest instability and residual deviations compared to the WT variant.

**TABLE 2:** Interpretation of MD simulation results.

Protein	RMSD (nm)	RMSF (nm)	Rg (nm)	SASA (nm^2^)	H‐BOND
WT	0.283	0.175	1.426	62.69	∼91
D108Y	0.229 ↓	0.163↓	1.428 ↑	63.91 ↑	∼98 ↑
C111Y	0.258 ↓	0.176↑	1.430 ↑	63.39 ↑	∼93 ↑
Y144N	0.310 ↑, σ	0.206 ↑, σ	1.424 ↓, σ	61.24 ↓, σ	∼94 ↑
L151P	0.266 ↓	0.169 ↓	1.425 ↓	65.90 ↑	∼96 ↑
WT‐NLG	0.164	0.141	1.500 σ	75.681	∼52
D108Y‐NLG	0.161 ↓	0.132 ↓	1.505 ↑	76.098 ↑	∼52 ↑
C111Y‐NLG	0.183 ↑	0.136 ↓	1.516 ↑	76.433 ↑	∼51 ↓
Y144N‐NLG	0.149 ↓	0.129 ↓	1.499 ↓	77.224 ↑	∼52 ↑
L151P‐NLG	0.159 ↓, σ	0.131 ↓, σ	1.508 ↑	78.400 ↑, σ	∼53 ↑
8CX3‐WT	0.361	0.170	1.818	102.469	∼145
8CX3‐D108Y	0.427 ↑	0.186 ↑	1.821 ↑	103.333 ↑	∼140 ↓
8CX3‐C111Y	0.835 ↑, σ	0.824 ↑, σ	1.957 ↑, σ	105.620 ↑	∼142 ↓
8CX3‐Y144N	0.324 ↓	0.195 ↑	1.814 ↓	103.306 ↑	∼143 ↓
8CX3‐L151P	0.272 ↓	0.160 ↓	1.796 ↓	101.947 ↓, σ	∼146 ↑

*Note:* Here, ‘↑’ = higher than wild‐type; ‘↓’ = lower than wild‐type; ‘σ’ = higher deviation.

## 5 Conclusion

In summary, this study extensively investigated missense nsSNPs in the MUC16 gene, prevalent in various cancers. Computational analysis identified 84 pathogenic mutations, with four SEA5 domain mutations exhibiting high conservation and affecting protein stability. Molecular dynamics simulations and docking studies underscored the significance of the Y144N mutation in cancer progression. However, this work has limitations, including the short 100 ns molecular dynamics timeframe and simplified simulation conditions lacking cellular complexity. Analyses were restricted to missense mutations in the SEA5 domain, excluding other potentially relevant regions. Interaction studies were based on static models, not accounting for dynamic glycosylation or tumour microenvironment factors. Additionally, the predicted functional impacts of mutations require experimental validation through biochemical and in vivo analysis. Despite these limitations, our investigational findings shed light on MUC16‐related cancers, paving the way for future research and potential therapeutic avenues.

## Author Contributions

**Md Afjalus Siraj:** conceptualization (lead), investigation (supporting), methodology (lead), resources (lead), supervision (lead), visualization (lead), writing – original draft (lead), writing – review and editing (lead). **Muaz Faruque:** data curation (equal), formal analysis (equal), investigation (equal), project administration (equal), visualization (equal), writing – original draft (equal), writing – review and editing (equal). **Maisha Maliha Medha:** data curation (equal), formal analysis (equal), investigation (equal), project administration (equal), writing – original draft (equal). **A. M. U. B. Mahfuz:** conceptualization (supporting), methodology (supporting), software (supporting), validation (supporting). **Md. Monirul Islam:** project administration (supporting), resources (supporting), software (lead).

## Conflicts of Interest

The authors assert that there are no conflicts of interest associated with the publication of this paper.

## Supporting information


Figure S1.

Table S1.

## Data Availability

Data will be available upon request.
