# Supplementary material for: Deciphering Deleterious nsSNPs in MUC16's SEA Domain: Structural and Functional Implications in Cancer Metastasis via Computational Analysis
Source: J Cell Mol Med. 2025 Jun 6;29(11):e70633. doi: 10.1111/jcmm.70633 (PMC12143179; doi:10.1111/jcmm.70633)
Supplement: Supplementary file 1 — Figure S1. [file JCMM-29-e70633-s001.pptx]

## Slide 1
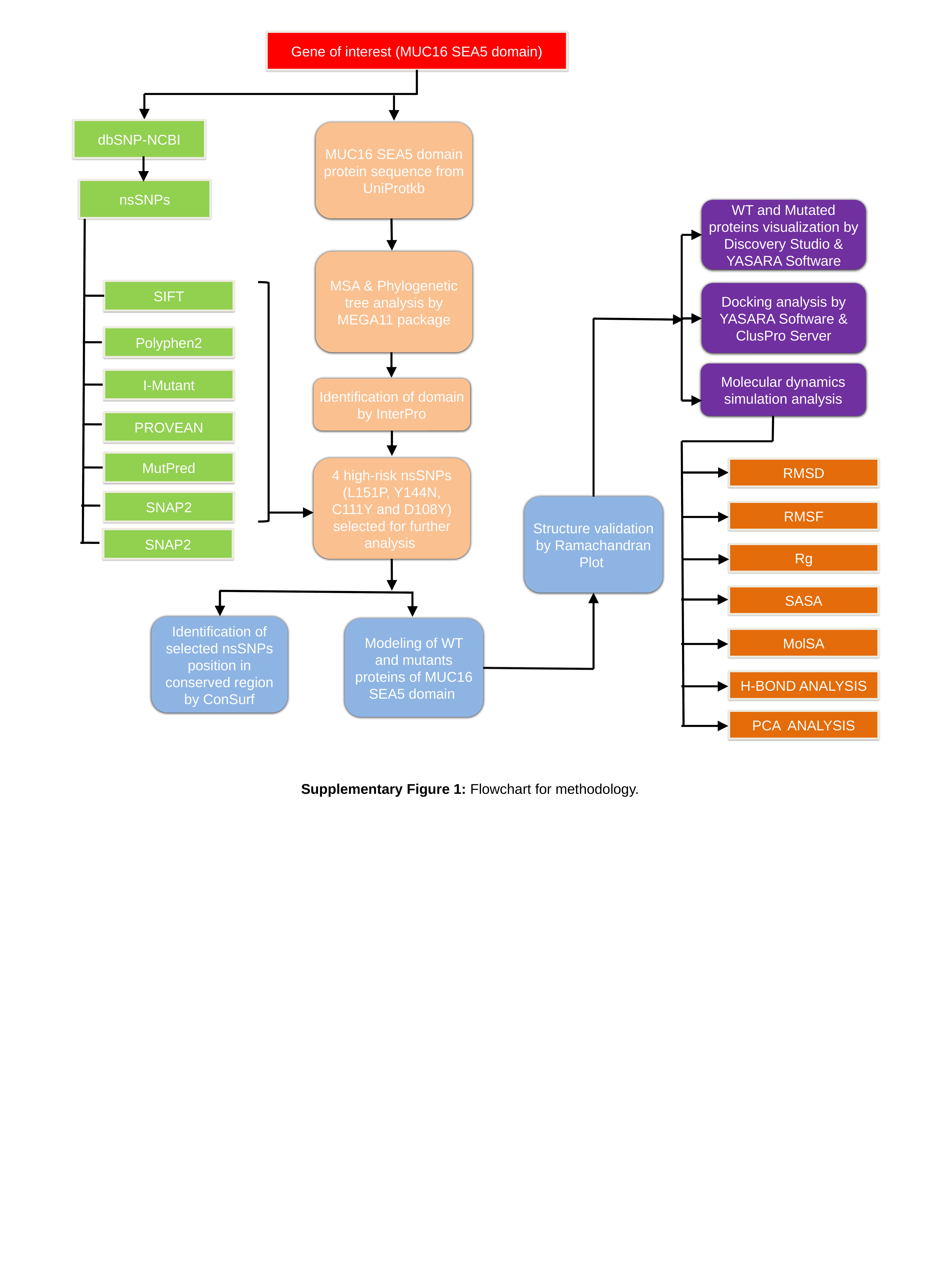

Gene of interest (MUC16 SEA5 domain)
dbSNP-NCBI
MUC16 SEA5 domain protein sequence from UniProtkb
nsSNPs
WT and Mutated proteins visualization by Discovery Studio & YASARA Software
MSA & Phylogenetic tree analysis by MEGA11 package
SIFT
Docking analysis by YASARA Software & ClusPro Server
Polyphen2
Molecular dynamics simulation analysis
I-Mutant
Identification of domain by InterPro
PROVEAN
MutPred
4 high-risk nsSNPs (L151P, Y144N, C111Y and D108Y) selected for further analysis
RMSD
SNAP2
Structure validation by Ramachandran Plot
RMSF
Rg
SASA
Identification of selected nsSNPs position in conserved region by ConSurf
Modeling of WT and mutants proteins of MUC16 SEA5 domain
MolSA
H-BOND ANALYSIS
PCA ANALYSIS
Supplementary Figure 1: Flowchart for methodology.
SNAP2

## Slide 2
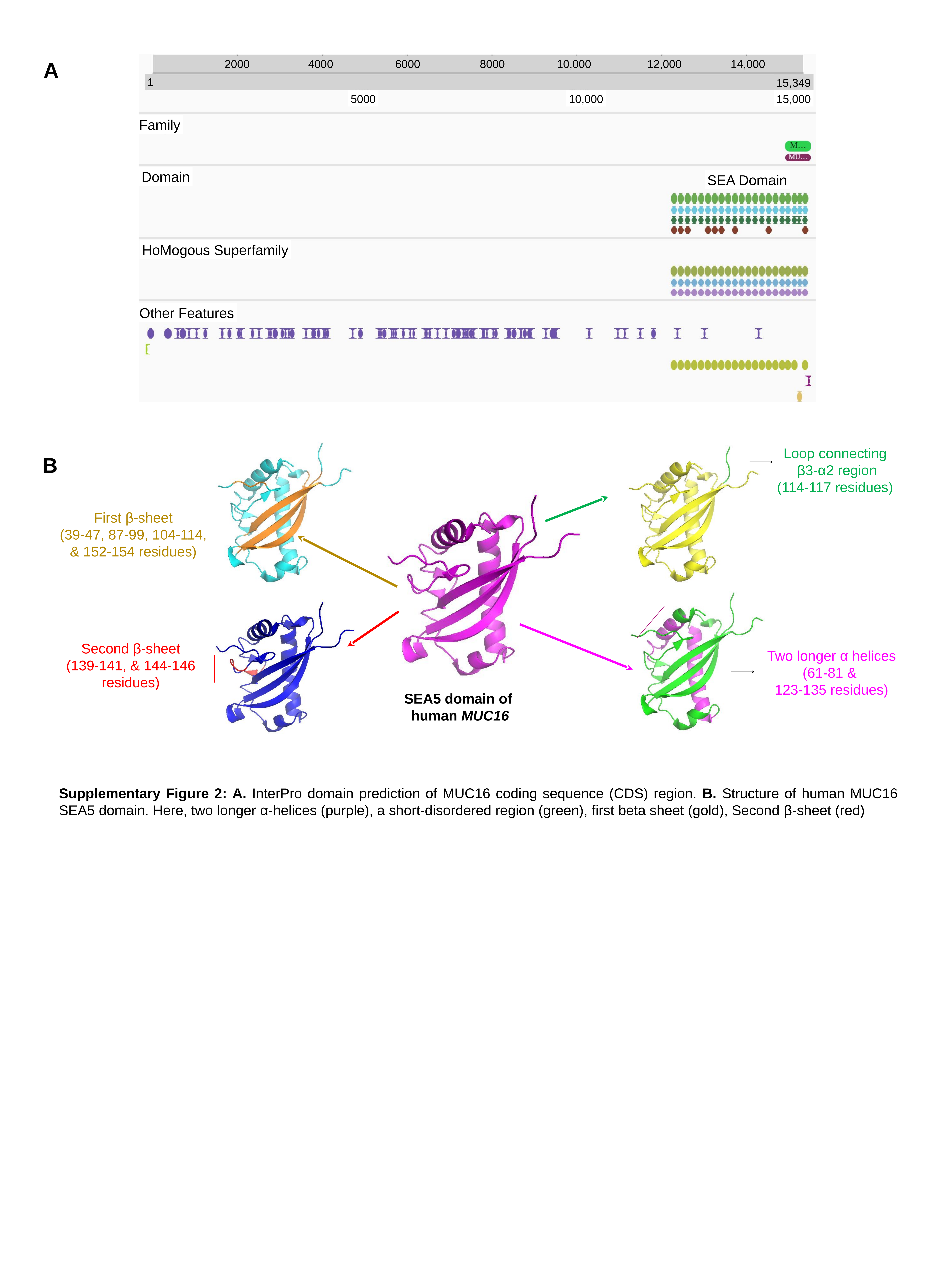

4000
6000
8000
10,000
14,000
2000
12,000
1
15,349
5000
10,000
15,000
Family
Domain
SEA Domain
HoMogous Superfamily
Other Features
A
First β-sheet
(39-47, 87-99, 104-114, & 152-154 residues)
Loop connecting
β3-α2 region
(114-117 residues)
Two longer α helices
(61-81 &
123-135 residues)
Second β-sheet
(139-141, & 144-146 residues)
SEA5 domain of
human MUC16
B
Supplementary Figure 2: A. InterPro domain prediction of MUC16 coding sequence (CDS) region. B. Structure of human MUC16 SEA5 domain. Here, two longer α-helices (purple), a short-disordered region (green), first beta sheet (gold), Second β-sheet (red)

## Slide 3
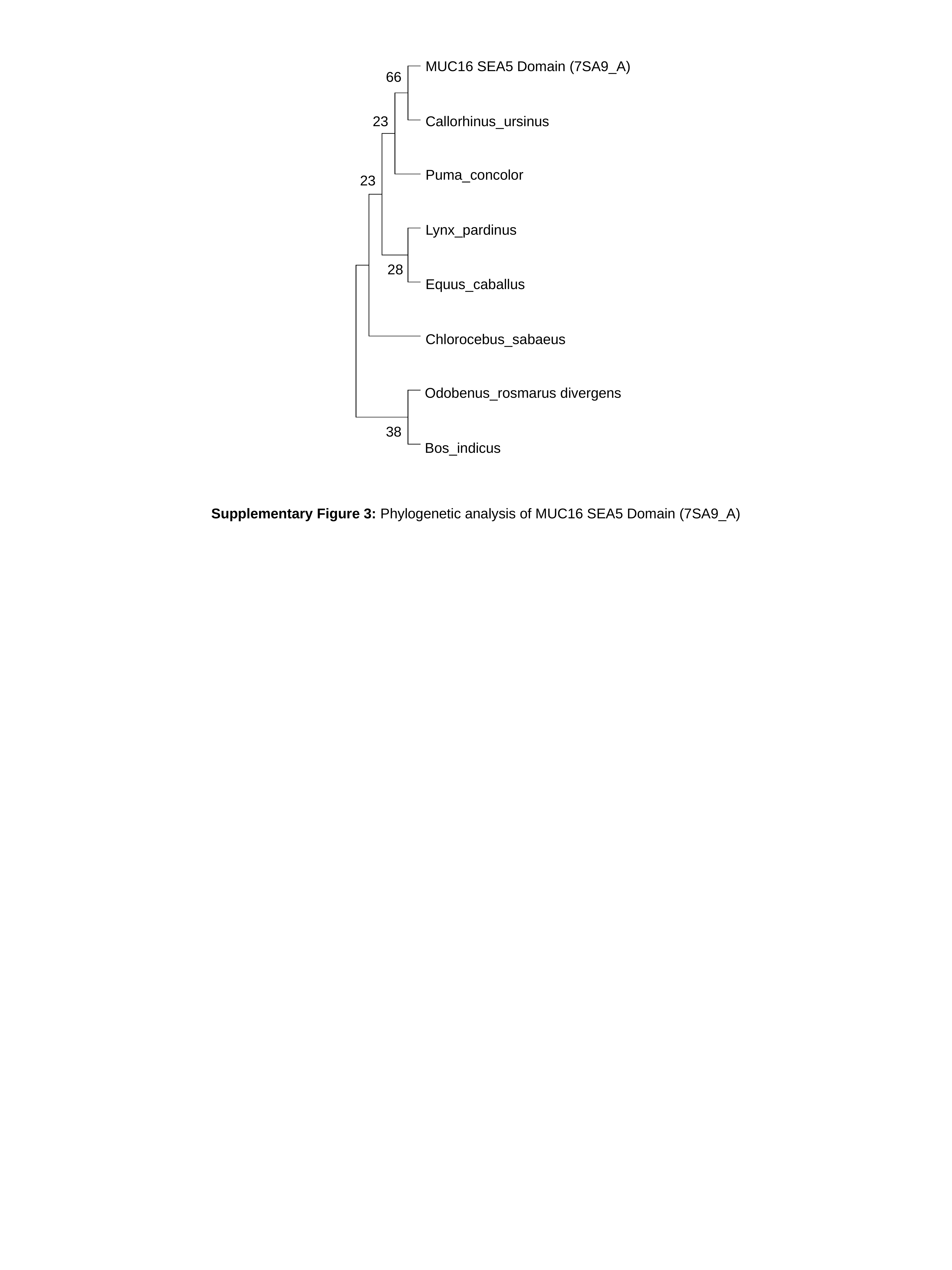

MUC16 SEA5 Domain (7SA9_A)
66
23
Callorhinus_ursinus
Puma_concolor
23
Lynx_pardinus
28
Equus_caballus
Chlorocebus_sabaeus
Odobenus_rosmarus divergens
38
Bos_indicus
Supplementary Figure 3: Phylogenetic analysis of MUC16 SEA5 Domain (7SA9_A)

## Slide 4
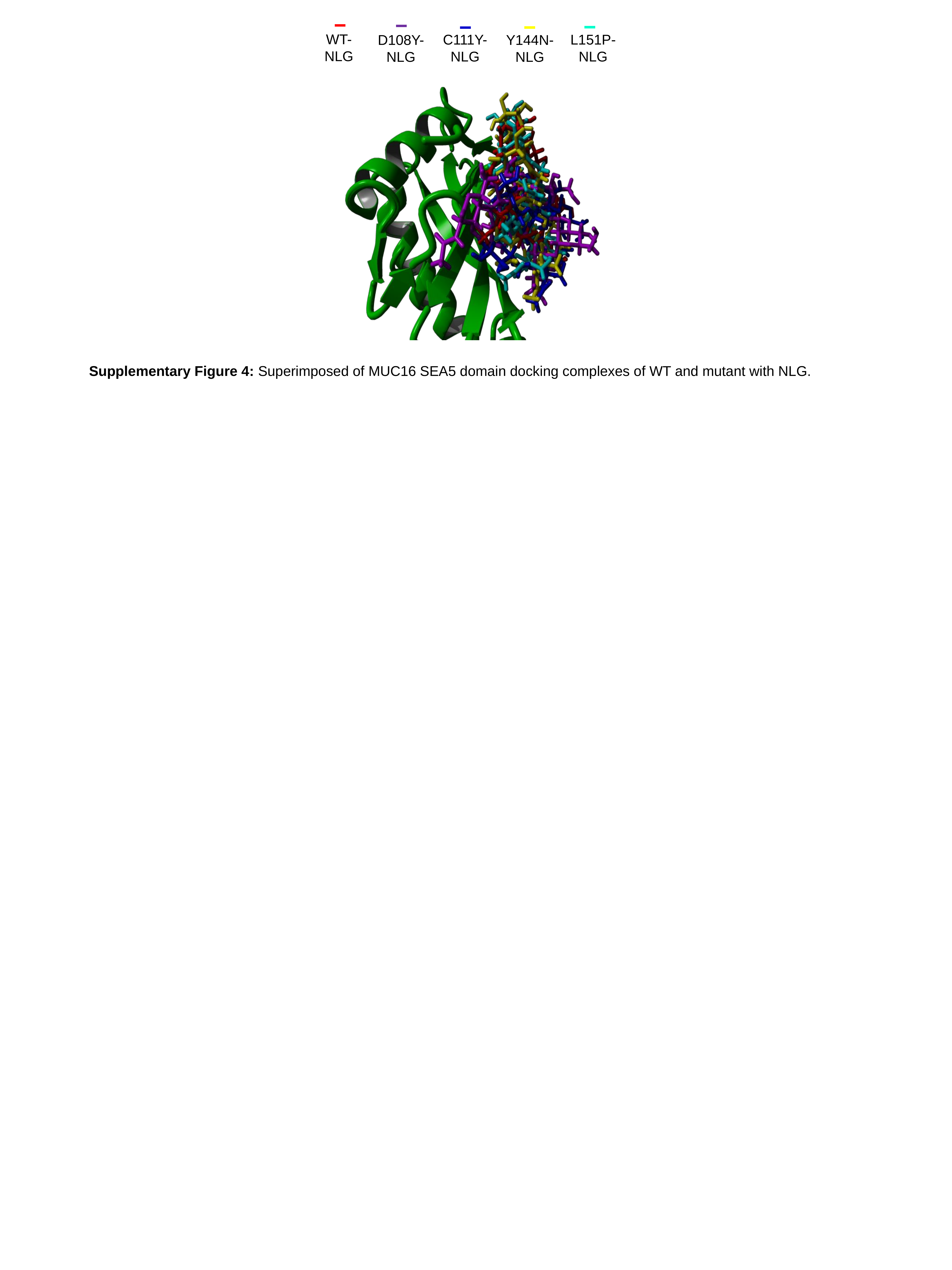

WT-
NLG
L151P-
NLG
C111Y-
NLG
D108Y-
NLG
Y144N-
NLG
Supplementary Figure 4: Superimposed of MUC16 SEA5 domain docking complexes of WT and mutant with NLG.

## Slide 5
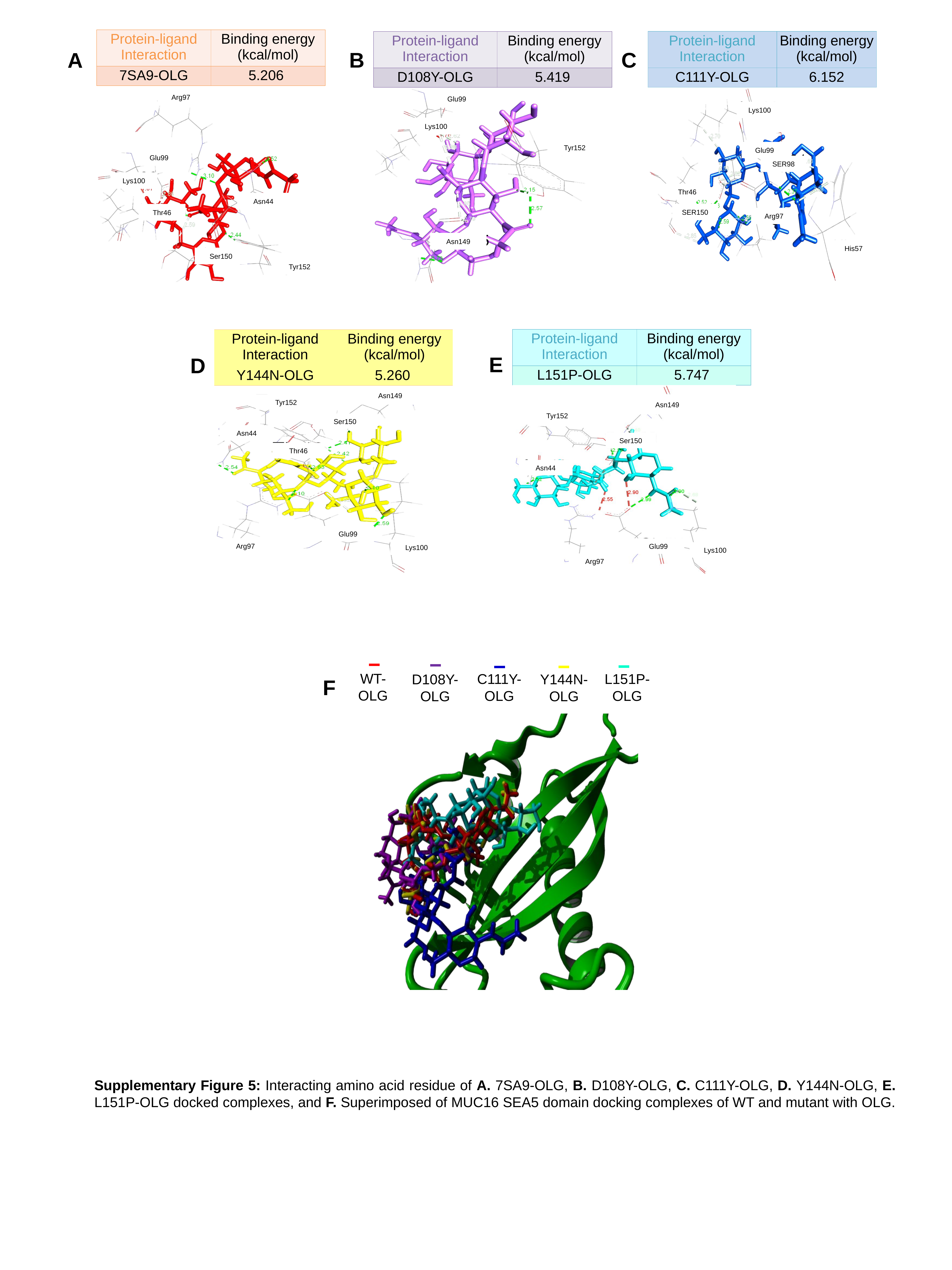

| Protein-ligand Interaction | Binding energy (kcal/mol) |
| --- | --- |
| 7SA9-OLG | 5.206 |
| Protein-ligand Interaction | Binding energy (kcal/mol) |
| --- | --- |
| C111Y-OLG | 6.152 |
| Protein-ligand Interaction | Binding energy (kcal/mol) |
| --- | --- |
| D108Y-OLG | 5.419 |
B
C
A
Arg97
Glu99
Lys100
Asn44
Thr46
Ser150
Tyr152
Glu99
Lys100
Tyr152
Asn149
Lys100
Glu99
SER98
Thr46
SER150
Arg97
His57
Asn149
Tyr152
Ser150
Asn44
Glu99
Lys100
Lys100
Arg97
Asn149
Tyr152
Ser150
Asn44
Thr46
Glu99
Arg97
Lys100
| Protein-ligand Interaction | Binding energy (kcal/mol) |
| --- | --- |
| L151P-OLG | 5.747 |
| Protein-ligand Interaction | Binding energy (kcal/mol) |
| --- | --- |
| Y144N-OLG | 5.260 |
E
D
WT-
OLG
L151P-
OLG
C111Y-
OLG
D108Y-
OLG
Y144N-
OLG
F
Supplementary Figure 5: Interacting amino acid residue of A. 7SA9-OLG, B. D108Y-OLG, C. C111Y-OLG, D. Y144N-OLG, E. L151P-OLG docked complexes, and F. Superimposed of MUC16 SEA5 domain docking complexes of WT and mutant with OLG.
