# Supplementary material for: Deciphering Deleterious nsSNPs in MUC16's SEA Domain: Structural and Functional Implications in Cancer Metastasis via Computational Analysis
Source: J Cell Mol Med. 2025 Jun 6;29(11):e70633. doi: 10.1111/jcmm.70633 (PMC12143179; doi:10.1111/jcmm.70633)
Supplement: Supplementary file 2 — Table S1. [file JCMM-29-e70633-s002.docx]

**SUPPLEMENTARY TABLES**

Deciphering Deleterious nsSNPs in MUC16’s SEA Domain: Structural and Functional Implications in Cancer Metastasis via Computational Analysis

### Muaz Faruque^1¥^, Maisha Maliha Medha^1¥^, A.M.U.B Mahfuz^2^, Md. Monirul Islam^1^,

### Md Afjalus Siraj^3*^

^1^ Pharmacy Discipline, Life Science School, Khulna University, Khulna-9208, Bangladesh.

^2^ Department of Biotechnology and Genetic Engineering, Faculty of Life Science, University of Development Alternative, Dhaka-1209, Bangladesh.

^3^ Department of Pharmacy, Faculty of Health Sciences, Gono Bishwabidyalay, Dhaka-1344, Bangladesh.

^¥^ These authors contributes equally.

***Corresponding author:**

Md Afjalus Siraj, Ph.D.

Department of Pharmacy, Faculty of Health Sciences, Gono Bishwabidyalay, Dhaka-1344, Bangladesh.

Email: [afjalus.siraj@gmail.com](mailto:afjalus.siraj@gmail.com)

**Supplementary Table 1:** Analysis of Ramachandran plot of modeled structures using PROCHECK server.

| Model | Residues in most favored regions | | Residues in additionally allowed regions | | Residue in generously allowed region | |
| --- | --- | --- | --- | --- | --- | --- |
|  | No. of residues | % of residues | No. of residues | % of residues | No. of residues | % of residues |
| WT | 98 | 92.5 | 7 | 6.6 | 1 | 0.9 |
| D108Y | 97 | 91.5 | 8 | 7.5 | 1 | 0.9 |
| C111Y | 97 | 91.5 | 8 | 7.5 | 1 | 0.9 |
| Y144N | 97 | 91.5 | 8 | 7.5 | 1 | 0.9 |
| L151P | 96 | 91.4 | 8 | 7.5 | 1 | 0.9 |

**Supplementary Table 2:** Non-bon interaction of MUC16 SEA5 domain wild-type and mutant proteins with NLG.

| Binding energy[kcal/mol]  of 7SA9-NLG | Interacting Residue | Distance (Å) | Bond  Type |
| --- | --- | --- | --- |
| 6.022  kcal/mol | ASN44 | 2.64433 | CH |
|  | ASN44 | 2.07468 | CH |
|  | TYR152 | 3.04013 | CH |
|  | GLN159 | 2.40636 | CH |
|  | THR46 | 3.0203 | CH |
|  | ASP108 | 2.44573 | CH |
|  | ASP147 | 2.66064 | CH |
|  | ASN154 | 2.42803 | CH |
|  | THR42 | 2.84202 | C |
|  | GLN159 | 2.81849 | C |
|  | TYR152 | 2.7914 | C |
|  | TYR152 | 2.17465 | C |
|  | ASN154 | 3.07615 | C |
|  | ASN44 | 2.52286 | C |
|  | TYR152 | 2.46087 | P |
|  | THR42 | 1.50085 | UDD |
|  | ASN44 | 2.11312 | UDD |

Here, Conventional Hydrogen Bond = CH, Carbon Hydrogen Bond = C, Pi-Sigma = P, Unfavorable Donor-Donor =UDD, Unfavorable Acceptor-Acceptor = UAA

| Binding energy[kcal/mol]  Of D108Y-NLG | Interacting Residue | Distance (Å) | Bond  Type |
| --- | --- | --- | --- |
| 6.225  kcal/mol | THR42 | 2.06912 | CH |
|  | ASN44 | 2.68133 | CH |
|  | ASN44 | 2.18361 | CH |
|  | ARG97 | 2.44079 | CH |
|  | TYR108 | 1.99847 | CH |
|  | ARG148 | 2.94484 | CH |
|  | ASN149 | 3.0922 | CH |
|  | GLU99 | 2.54824 | CH |
|  | GLU99 | 2.07978 | CH |
|  | ASN154 | 2.74924 | CH |
|  | ALA109 | 1.86519 | CH |
|  | SER98 | 2.46077 | CH |
|  | SER150 | 2.76033 | C |
|  | SER150 | 3.04329 | C |
|  | SER150 | 2.51145 | C |
|  | ASN149 | 2.41682 | C |
|  | LEU43 | 2.47892 | UDD |

Here, Conventional Hydrogen Bond = CH, Carbon Hydrogen Bond = C, Pi-Sigma = P, Unfavorable Donor-Donor =UDD, Unfavorable Acceptor-Acceptor = UAA

| Binding energy[kcal/mol]  of C111Y-NLG | Interacting Residue | Distance (Å) | Bond Type |
| --- | --- | --- | --- |
| 5.818  kcal/mol | THR42 | 2.35442 | CH |
|  | ASN44 | 1.85456 | CH |
|  | THR94 | 2.71837 | CH |
|  | ARG97 | 2.51615 | CH |
|  | ARG97 | 2.95999 | CH |
|  | ARG97 | 2.51783 | CH |
|  | LYS100 | 2.5395 | C |
|  | LYS100 | 2.68641 | C |
|  | TYR152 | 2.6115 | C |
|  | ASP108 | 2.65233 | C |
|  | ASN44 | 2.35445 | C |
|  | ASN149 | 2.59048 | C |
|  | ASN149 | 1.99502 | C |
|  | GLU99 | 2.36075 | C |
|  | ASP147 | 1.79709 | C |
|  | GLU99 | 2.22514 | C |
|  | LEU43 | 2.88399 | C |
|  | THR42 | 2.58282 | C |
|  | LYS100 | 2.67975 | C |
|  | ASN44 | 3.04293 | C |
|  | ASN149 | 2.80061 | C |
|  | ASN44 | 2.61985 | C |
|  | ALA109 | 2.87417 | C |
|  | THR46 | 1.67122 | UDD |
|  | ARG97 | 1.76894 | UDD |
|  | ALA109 | 1.5777 | UDD |
|  | GLU99 | 2.90784 | UAA |

Here, Conventional Hydrogen Bond = CH, Carbon Hydrogen Bond = C, Pi-Sigma = P, Unfavorable Donor-Donor =UDD, Unfavorable Acceptor-Acceptor = UAA

| Binding energy[kcal/mol]  of Y144N-NLG | Interacting Residue | Distance (Å) | Bond  Type |
| --- | --- | --- | --- |
| 6.253  kcal/mol | THR42 | 1.90544 | CH |
|  | ARG97 | 2.91036 | CH |
|  | ARG97 | 2.50569 | CH |
|  | ARG97 | 2.04982 | CH |
|  | TYR152 | 2.3122 | CH |
|  | ASP108 | 2.92102 | CH |
|  | ASN149 | 2.00396 | CH |
|  | THR157 | 2.15961 | CH |
|  | GLU99 | 2.02883 | CH |
|  | ASN154 | 2.07892 | CH |
|  | SER150 | 2.85286 | C |
|  | ASN149 | 3.01147 | C |
|  | TYR152 | 2.79239 | UAA |
|  | GLY155 | 2.85851 | UAA |

Here, Conventional Hydrogen Bond = CH, Carbon Hydrogen Bond = C, Pi-Sigma = P, Unfavorable Donor-Donor =UDD, Unfavorable Acceptor-Acceptor = UAA

| Binding energy[kcal/mol]  of L151P-NLG | Interacting Residue | Distance (Å) | Bond  Type |
| --- | --- | --- | --- |
| 6.405  kcal/mol | ASN44 | 2.17087 | CH |
|  | ARG97 | 2.83934 | CH |
|  | ARG97 | 2.67809 | CH |
|  | ARG97 | 3.02827 | CH |
|  | THR157 | 2.07624 | CH |
|  | GLU99 | 2.44981 | CH |
|  | GLU99 | 2.97499 | CH |
|  | ASN44 | 2.44132 | CH |
|  | ASN149 | 2.42589 | CH |
|  | THR157 | 2.1382 | CH |
|  | GLY155 | 1.79967 | CH |
|  | GLY155 | 2.00441 | CH |
|  | ASN149 | 2.24974 | CH |
|  | SER150 | 2.55319 | C |
|  | THR157 | 2.96281 | C |
|  | ASN44 | 2.68129 | C |
|  | THR42 | 1.21784 | UDD |
|  | ARG97 | 1.65067 | UDD |
|  | THR157 | 2.47628 | UDD |
|  | TYR152 | 2.80077 | UAA |
|  | GLN159 | 2.63374 | UAA |

Here, Conventional Hydrogen Bond = CH, Carbon Hydrogen Bond = C, Pi-Sigma = P, Unfavorable Donor-Donor =UDD, Unfavorable Acceptor-Acceptor = UAA

**Supplementary Table 3:** Non-bon interaction of MUC16 SEA5 domain wild-type and mutant proteins with OLG.

| Binding energy[kcal/mol]  of 7SA9-OLG | Interacting Residue | Distance (Å) | Bond  Type |
| --- | --- | --- | --- |
| 5.206  kcal/mol | ASN44 | 2.51771 | CH |
|  | THR46 | 2.63368 | CH |
|  | ARG97 | 3.09697 | CH |
|  | TYR152 | 2.44107 | CH |
|  | SER150 | 2.59434 | C |
|  | GLU99 | 2.86151 | C |
|  | LYS150 | 1.81432 | UDD |
|  | GLU99 | 2.70618 | UAA |

Here, Conventional Hydrogen Bond = CH, Carbon Hydrogen Bond = C, Pi-Sigma = P, Unfavorable Donor-Donor =UDD, Unfavorable Acceptor-Acceptor = UAA

| Binding energy[kcal/mol]  Of D108Y-OLG | Interacting Residue | Distance (Å) | Bond  Type |
| --- | --- | --- | --- |
| 5.419  kcal/mol | ASN149 | 2.73346 | CH |
|  | TYR152 | 2.14735 | CH |
|  | TYR152 | 2.57288 | CH |
|  | ASN149 | 2.7894 | C |
|  | ASN149 | 2.64761 | C |
|  | GLU99 | 3.00327 | C |
|  | GLU99 | 2.62195 | C |
|  | GLU99 | 2.97779 | C |
|  | LYS100 | 1.75948 | UDD |

Here, Conventional Hydrogen Bond = CH, Carbon Hydrogen Bond = C, Pi-Sigma = P, Unfavorable Donor-Donor =UDD, Unfavorable Acceptor-Acceptor = UAA

| Binding energy[kcal/mol]  of C111Y-OLG | Interacting Residue | Distance (Å) | Bond Type |
| --- | --- | --- | --- |
| 6.152  kcal/mol | ARG97 | 2.29693 | CH |
|  | ARG97 | 2.54675 | CH |
|  | ARG97 | 2.59258 | CH |
|  | SER98 | 2.3705 | CH |
|  | THR46 | 2.52179 | CH |
|  | GLU99 | 2.95982 | CH |
|  | HIS57 | 2.87668 | C |
|  | SER98 | 2.63498 | C |
|  | GLU99 | 2.56854 | C |
|  | LYS100 | 2.70402 | C |
|  | SER98 | 2.46361 | C |
|  | GLU99 | 2.81724 | C |
|  | GLU99 | 2.53157 | C |
|  | GLU99 | 2.20585 | C |
|  | SER150 | 2.85367 | C |

Here, Conventional Hydrogen Bond = CH, Carbon Hydrogen Bond = C, Pi-Sigma = P, Unfavorable Donor-Donor =UDD, Unfavorable Acceptor-Acceptor = UAA

| Binding energy[kcal/mol]  of Y144N-OLG | Interacting Residue | Distance (Å) | Bond  Type |
| --- | --- | --- | --- |
| 5.260  kcal/mol | ASN44 | 2.53735 | CH |
|  | THR46 | 2.63257 | CH |
|  | ARG97 | 3.09616 | CH |
|  | LYS100 | 2.58968 | CH |
|  | LYS100 | 2.09752 | CH |
|  | TYR152 | 2.41584 | CH |
|  | TYR152 | 2.40922 | CH |
|  | ASN149 | 2.87399 | CH |
|  | SER150 | 2.57867 | C |
|  | GLU99 | 3.01537 | C |

Here, Conventional Hydrogen Bond = CH, Carbon Hydrogen Bond = C, Pi-Sigma = P, Unfavorable Donor-Donor =UDD, Unfavorable Acceptor-Acceptor = UAA

| Binding energy[kcal/mol]  Of L151P-OLG | Interacting Residue | Distance (Å) | Bond  Type |
| --- | --- | --- | --- |
| 5.747  kcal/mol | ASN44 | 2.52432 | CH |
|  | LYS100 | 2.90244 | CH |
|  | TYR152 | 2.18267 | CH |
|  | TYR152 | 2.13218 | CH |
|  | GLU99 | 2.98934 | CH |
|  | LYS100 | 2.67743 | C |
|  | SER150 | 2.73467 | C |
|  | ASN149 | 2.39586 | C |
|  | ARG97 | 2.5527 | UDD |
|  | GLU99 | 2.89905 | UAA |

Here, Conventional Hydrogen Bond = CH, Carbon Hydrogen Bond = C, Pi-Sigma = P, Unfavorable Donor-Donor =UDD, Unfavorable Acceptor-Acceptor = UAA

**Supplementary Table 4:** Non-bond interaction of mesothelin with MUC16 SEA5 domain wild-type and mutant proteins.

| Weighted Score  of 8CX3-7SA9 | Interacting Residue | Distance (Å) | Bond  Type |
| --- | --- | --- | --- |
| -929.3 | ARG69-GLU347 | 2.01128 | SB |
|  | LYS80-GLU313 | 1.84308 | SB |
|  | LYS80-GLU313 | 1.75516 | SB |
|  | ARG114-ASP337 | 1.86898 | SB |
|  | ARG114-ASP337 | 1.78235 | SB |
|  | TYR346-ASN65 | 1.99686 | CH |
|  | ARG69-THR345 | 1.88355 | CH |
|  | GLN72-PRO343 | 1.88115 | CH |
|  | CYS91-ALA341 | 2.40167 | CH |
|  | ARG92-PHE344 | 1.75472 | CH |
|  | THR345-GLU68 | 3.69727 | C |

Here, Salt Bridge = SB, Conventional Hydrogen Bond = CH, Carbon Hydrogen Bond = C, Pi-Sigma = P, Unfavorable Donor-Donor =UDD, Unfavorable Acceptor-Acceptor = UAA

| Weighted Score  Of 8CX3-D108Y | Interacting Residue | Distance (Å) | Bond  Type |
| --- | --- | --- | --- |
| -945.4 | ARG69-GLU347 | 1.87776 | SB |
|  | LYS80-GLU313 | 1.84769 | SB |
|  | LYS80-GLU313 | 1.804 | SB |
|  | ARG114-ASP337 | 1.82126 | SB |
|  | ARG114-ASP337 | 1.78717 | SB |
|  | THR345-GLN72 | 1.89142 | CH |
|  | LYS378-PRO59 | 1.8432 | CH |
|  | ARG69-THR345 | 2.42819 | CH |
|  | ARG69-THR345 | 1.86446 | CH |
|  | ARG92-PHE344 | 1.65919 | CH |
|  | ARG92-PHE344 | 2.73286 | CH |
|  | THR345-GLU68 | 3.36346 | C |

Here, Salt Bridge = SB, Conventional Hydrogen Bond = CH, Carbon Hydrogen Bond = C, Pi-Sigma = P, Unfavorable Donor-Donor =UDD, Unfavorable Acceptor-Acceptor = UAA

| Weighted Score  of 8CX3-C111Y | Interacting Residue | Distance (Å) | Bond Type |
| --- | --- | --- | --- |
| -941.0 | LYS299-GLU54 | 1.73211 | SB |
|  | LYS299-GLU54 | 2.15789 | SB |
|  | LYS320-ASP101 | 1.87075 | SB |
|  | LYS320-ASP101 | 1.74702 | SB |
|  | ARG97-GLU347 | 1.90311 | SB |
|  | ARG97-GLU347 | 2.77541 | SB |
|  | LYS66-GLU347 | 1.73542 | SB |
|  | LYS320-SER98 | 1.79848 | CH |
|  | THR345-ASP108 | 1.9021 | CH |
|  | LYS378-ASN149 | 1.65493 | CH |
|  | LYS378-GLN159 | 1.71898 | CH |
|  | ASN44-PRO343 | 2.75787 | CH |
|  | ASN44-PRO343 | 2.58479 | CH |
|  | ARG124-TYR374 | 2.05732 | CH |
|  | ARG124-ASN340 | 2.13684 | CH |
|  | ARG124-TYR374 | 1.87437 | CH |
|  | ARG120-ASN340 | 1.81247 | CH |
|  | TRP129-HIS405 | 2.17007 | CH |

Here, Salt Bridge = SB, Conventional Hydrogen Bond = CH, Carbon Hydrogen Bond = C, Pi-Sigma = P, Unfavorable Donor-Donor =UDD, Unfavorable Acceptor-Acceptor = UAA

| Weighted Score  Of 8CX3-Y144N | Interacting Residue | Distance (Å) | Bond  Type |
| --- | --- | --- | --- |
| -926.0 | ARG69-GLU347 | 2.00076 | SB |
|  | LYS80-GLU313 | 1.8534 | SB |
|  | LYS80-GLU313 | 1.80135 | SB |
|  | ARG114-ASP337 | 1.87241 | SB |
|  | ARG114-ASP337 | 1.78084 | SB |
|  | TYR346-ASN65 | 2.00352 | CH |
|  | ARG69-THR345 | 1.85513 | CH |
|  | GLN72-GLN348 | 2.68328 | CH |
|  | GLN72-PRO343 | 1.85996 | CH |
|  | CYS91-ALA341 | 2.40951 | CH |
|  | ARG98-PHE344 | 1.75235 | CH |
|  | THR345-GLU68 | 3.69501 | C |

Here, Salt Bridge = SB, Conventional Hydrogen Bond = CH, Carbon Hydrogen Bond = C, Pi-Sigma = P, Unfavorable Donor-Donor =UDD, Unfavorable Acceptor-Acceptor = UAA

| Weighted Score  Of 8CX3-L151P | Interacting Residue | Distance (Å) | Bond  Type |
| --- | --- | --- | --- |
| -916.1 | ARG69-GLU347 | 2.1818 | SB |
|  | LYS54-GLU313 | 1.8332 | SB |
|  | LYS54-AGLU313 | 1.7458 | SB |
|  | ARG114-ASP337 | 1.8721 | SB |
|  | ARG114-ASP337 | 1.7831 | SB |
|  | TYR346-ASN65 | 1.998 | CH |
|  | ARG69-THR345 | 1.7675 | CH |
|  | GLN72-PRO343 | 2.0107 | CH |
|  | CYS91-ALA341 | 2.3297 | CH |
|  | ARG92-PHE344 | 1.955 | CH |

Here, Salt Bridge = SB, Conventional Hydrogen Bond = CH, Carbon Hydrogen Bond = C, Pi-Sigma = P, Unfavorable Donor-Donor =UDD, Unfavorable Acceptor-Acceptor = UAA
